# The emergence of ecotypes in a parasitoid wasp: a case of incipient sympatric speciation in Hymenoptera?

**DOI:** 10.1186/s12862-021-01938-y

**Published:** 2021-11-15

**Authors:** Pawel Malec, Justus Weber, Robin Böhmer, Marc Fiebig, Denise Meinert, Carolin Rein, Ronja Reinisch, Maik Henrich, Viktoria Polyvas, Marie Pollmann, Lea von Berg, Christian König, Johannes L. M. Steidle

**Affiliations:** 1 Naturpark Steigerwald E.V., 91443 Scheinfeld, Germany; 2 Dep. of Chemical Ecology 190T, Institute of Biology, University of Hohenheim, 70593 Stuttgart, Germany; 3 Natural History Museum Bern, 3005 Bern, Switzerland; 4 Untere Naturschutzbehörde, Landratsamt Kitzingen, 97318 Kitzingen, Germany; 5 Kerschensteiner Schule, 70469 Stuttgart, Germany; 6 Apicultural State Institute, University of Hohenheim, 70593 Stuttgart, Germany; 7 Wildlife Ecology and Management, University of Freiburg, 79106 Freiburg, Germany; 8 Akademie für Natur- und Umweltschutz Baden-Württemberg beim Ministerium für Umwelt, Klima und Energiewirtschaft, 70192 Stuttgart, Germany

**Keywords:** Reproductive barriers, Sympatric speciation, Ecotypes, Premating barrier, Postmating barrier, Ecological speciation, Inbreeding

## Abstract

**Background:**

To understand which reproductive barriers initiate speciation is a major question in evolutionary research. Despite their high species numbers and specific biology, there are only few studies on speciation in Hymenoptera. This study aims to identify very early reproductive barriers in a local, sympatric population of *Nasonia vitripennis* (Walker 1836), a hymenopterous parasitoid of fly pupae. We studied ecological barriers, sexual barriers, and the reduction in F1-female offspring as a postmating barrier, as well as the population structure using microsatellites.

**Results:**

We found considerable inbreeding within female strains and a population structure with either three or five subpopulation clusters defined by microsatellites. In addition, there are two ecotypes, one parasitizing fly pupae in bird nests and the other on carrion. The nest ecotype is mainly formed from one of the microsatellite clusters, the two or four remaining microsatellite clusters form the carrion ecotype. There was slight sexual isolation and a reduction in F1-female offspring between inbreeding strains from the same microsatellite clusters and the same ecotypes. Strains from different microsatellite clusters are separated by a reduction in F1-female offspring. Ecotypes are separated only by ecological barriers.

**Conclusions:**

This is the first demonstration of very early reproductive barriers within a sympatric population of Hymenoptera. It demonstrates that sexual and premating barriers can precede ecological separation. This indicates the complexity of ecotype formation and highlights the general need for more studies within homogenous populations for the identification of the earliest barriers in the speciation process.

**Supplementary Information:**

The online version contains supplementary material available at 10.1186/s12862-021-01938-y.

## Background

To understand which barriers initiate speciation is one of the main questions of evolutionary research [1, 2]. For many decades it was assumed that geographic isolation of populations is the essential first step for speciation. Speciation in sympatry, i.e. speciation without geographic isolation, was considered to occur only in special cases like polyploidy [3] or hybrid speciation [4], but was excluded as main speciation mechanism, because gene flow should counteract selection [5]. Only by the end of the last century the concept of sympatric speciation became increasingly accepted [6, 7]. By then, compelling examples of sister taxa with substantial reproductive isolation have accumulated, that must have emerged in overlapping geographic ranges without an allopatric phase, according to the criteria defined by [1]. Now, the debate has shifted to the question, how frequently sympatric speciation occurs [7].

While allopatric speciation is probably driven by incompatibilities according to the Dobzhansky–Muller model [8], and mutation order processes [9], there are numerous theoretic models on sympatric speciation [6]. The dominant idea is that disruptive natural selection leads to assortative mating and reproductive isolation between subpopulations [1, 7]. Prominent examples are ecologically divergent fish [10, 11] or phytophagous insects, which separated via host shift [12, 13]. Specifically in phytophagous insects, sympatric host races, i.e. intermediate stages between polymorphic populations and full species [14], are taken as evidence that genetically differentiated populations can exist despite gene flow which indicates that sympatric speciation is common [12, 14]. As an alternative to natural selection, it is also assumed that disruptive sexual selection, e.g. runaway sexual selection processes [15] or sexual conflict [16] might lead to separation of subpopulations in sympatry. However, because evidence so far indicates that this process is often the consequence of an interaction between sexual and natural selection [17–21] (but also see [22]) it was suggested to drop sexual selection as unique speciation mechanism [21].

The Hymenoptera (bees, wasps and ants) occur in almost all terrestrial ecosystems in large species numbers where they often play a fundamental ecological role as herbivores, pollinators, or natural enemies [23]. They comprise about 153.088 species [24], which is about 8% of all described species [25]. The species richness within the Hymenoptera is mostly due to the monophyletic group of parasitoid wasps [26]. These reproduce by laying their eggs on or in their hosts, mostly other insects, which are consumed and killed by the developing parasitoid larvae [27, 28]. Currently, there are about 90.000 described species of parasitoid wasps (calculated according to data from [24, 29]). However, parasitoid wasps are heavily understudied due to their typical small size, which hampered traditional taxonomic studies based on morphology. Therefore, the actual number is probably much higher. Cryptic species are abundant [30] and new species are continuously being discovered (e.g. [31–36]). Based on the observation that most parasitoids are very host specific and almost all holometabolous insect species are attacked by at least one or even several parasitoid wasp species, [29] calculated that parasitoid wasps comprise between 833,000 to 1,107,487 species. This makes Hymenoptera the most species-rich order of all animals.

The reason for the high species diversity in parasitoid wasps is unclear. A comparison of the diversity in carnivorous parasitic insect lineages with their non-parasitic sister groups found no indication that parasitic insects diversify more rapidly than predatory, saprophagous, or phytophagous insects. Therefore the authors concluded that there is no evidence that parasitism itself is the cause of the spectacular diversity of parasitic Hymenoptera [37]. Obviously, other traits must have promoted speciation in this group. Already in 1968, Askew hypothesized for the largest parasitoid superfamily Chalcidoidea with 24.788 described species [24] and an estimated number of 500.000 species [38], that one key factor for their high speciation rate and high species numbers is inbreeding, i.e. mating between siblings [39]. He suggested that it could increase reproductive isolation of lineages by genetic drift [40, 41], and promote speciation similar to geographic barriers in allopatric populations. Interestingly, this hypothesis is analogous to the idea that the high diversity in the ambrosia beetle tribe Xyleborini results from haplodiploidy and reduced gene flow between lineages due to close inbreeding [42]. While inbreeding can cause severe fitness reductions in many hymenopterous species having single-locus complementary sex determination (sl-CSD), which is ancestral in Hymenoptera [43], it is frequent in Chalcidoidea which miss sl-CSD [44] and where deleterious alleles are eliminated in haploid males [45]. In Chalcidoidea, females often place most or all of their eggs in one host or host patch and the emerging offspring readily mate with each other [27, 28, 39]. In addition, many species are monandrous, i.e. females mate only once [46, 47] and a second mating with non-related males after dispersal from the natal patch is rare. As an adaptation to inbreeding, females produce offspring with a strongly female biased sex ratio to avoid competition between their male offspring [48, 49]. The hypothesis that inbreeding is a major cause for the diversity of Chalcidoidea was formulated already in 1968 and is regularly cited in the relevant literature on speciation of Hymenoptera or parasitoid wasps [27, 28, 50–52]. However, it has never been explicitly studied. Specifically in Chalcidoidea it is unclear if very early reproductive barriers between sympatric inbreeding female lines emerge before any other barriers, such as ecological separation, as would be predicted by the hypothesis.

Despite their high species numbers and their specific biology, there are only few studies on speciation and the evolution of reproductive barriers in Hymenoptera. So far, sympatric speciation was convincingly demonstrated only for three braconid species [53–56]. The most detailed studies on the evolution of reproductive barriers exist for the genera *Lariophagus* (Chalcidoidea: Pteromalidae)*,* parasitoids of beetle larvae [52, 57, 58], and *Nasonia* (Chalcidoidea: Pteromalidae), parasitoids of fly pupae in bird nests or on carrion [59–65]. Because the full genome is sequenced for three *Nasonia* species [66], this genus serves as a model for other biological traits, e.g. wing morphology [67], circadian rhythm and diapause [68, 69], chemical communication [64, 70], sex determination [71], evolution of venom [72], and learning [73, 74]. Reproductive barriers have been studied in *Nasonia* only at the species level [61, 75–78]. They are based mainly on genetic incompatibilities in nuclear-nuclear or nuclear-mitochondrial interactions according to the Dobzhansky–Muller model [59, 62, 79], and on endosymbiont induced cytoplasmic incompatibility (CI). CI is assumed to have initiated the separation between *N. longicornis* and *N. giraulti* [60] and represents the main reproductive barrier between all *Nasonia*-species in general [80]. While CI in *Nasonia* is caused by different strains of *Wolbachia*, which is the best studied reproductive manipulating endosymbiont [81], there are at least three other endosymbionts which are able to cause the same effect [82–85]. In crosses between infected males and uninfected females (unidirectional), or between males and females infected with different bacterial strains (bidirectional) all these endosymbionts cause mortality of the fertilized egg (female mortality type) or, in haplodiploid Hymenoptera, the conversion of a diploid female egg into a haploid male egg (male development type) [86]. In addition, CI can also be caused by a higher endosymbiont density in male spermatocytes as compared to the female egg [87].

So far, there are no studies on reproductive barriers within one species of the *Nasonia* complex, e.g. on the level of ecotypes. For *N. vitripennis* (Walker 1836), evidence for the existence of two sympatric ecotypes with small molecular differences using RAPD-markers and some reproductive isolation due to a reduction in F1-hybrid females and hybrid breakdown in the F2-generation was provided by [88]. One ecotype was assumed to parasitize fly pupae in bird nests, mostly *Protocalliphora* flies which are parasitic on nestlings but also carrion flies feeding on dead nestlings [89–92]. The other ecotype attacks only carrion flies (genera *Calliphora*, *Lucilia* etc.) next to carrion [93]. This suggests that ecological separation in sympatry plays a role in divergence in *N. vitripennis*. However, detailed studies on these ecotypes are missing and it is unclear whether ecological separation is in fact the first barrier to arise. As described above for Chalcidoidea parasitoids in general, inbreeding is common in *N. vitripennis*. Females mate only once, mostly with their brothers directly after emergence from the host, or even within the host [94]. While males are brachypterous, cannot fly and therefore stay at the natal patch [95], females disperse after mating to locate new host patches, thereby covering distances up to at least 2 km [96]. Most females parasitize hosts only in one patch [97] and lay usually between 15 and 30 eggs per host [98] and up to 800 eggs in total within their lifetime [95] of about 2 to 3 weeks [99].

We studied early barriers within a population of *N. vitripennis* to elucidate the very early barriers of separation leading to ecotype formation and speciation. To exclude the influence of geography, we studied the population within the area of a local park, i.e. in sympatry according to most definitions [5, 100]. Ecological isolation as a premating barrier was studied with respect to phenology, differences in the reaction to olfactory host habitat cues, and host acceptance. These are important steps for successful reproduction in parasitoids [101, 102]. In addition, we studied premating sexual isolation in mating experiments, and postmating isolation by assessing F1-female offspring within and between inbreeding strains and ecotypes. To address genetic divergence within the population, we also studied the population structure using microsatellites.

## Results

### Parasitization in the field

**Fig. 1:**
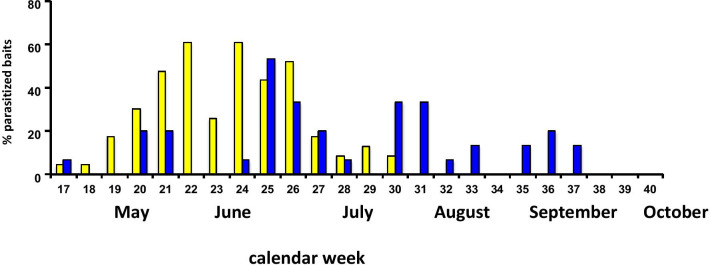
Percentage of *Lucilia sericata* baits that had been parasitized by *Nasonia vitripennis* females in bird nests (yellow) and next to carrion (blue) over a sampling period of 24 weeks (end of April–mid of October 2012) in the Hohenheim Park

Bait bags with fly pupae as hosts for *N. vitripennis* were exposed in the 35 ha large area of Hohenheim Park (Stuttgart, Germany) in bird nest boxes and next to small carrion hidden on the ground to attract wasps for parasitization. After 1 week, fly pupae were taken to the laboratory where wasps emerged, indicating parasitization in the field. Fly pupae exposed in bird nests, parasitization started in April, increased until the end of May and dropped until the end of July (Fig. 1). On carrion, parasitization occurred during the whole sampling period until September with some breaks in between, probably due to adverse weather conditions. Host pupae from control baits that were attached to twigs without surrounding nest material or carrion never yielded any *N. vitripennis* offspring.

### Ecological isolation

Wasps that emerged from host pupae, which were exposed in the field in the bait bags (see above), were used to establish laboratory strains for experiments. For each strain, only wasps emerging from one host pupa were taken into culture. Because wasps emerging from one host pupa are the offspring from one or two females [97], these strains simulated the natural situation in which offspring emerging from one host are inbreeding [97], but still maintain at least some genetic diversity. Up to the experiments, all strains were reared under identical conditions in the laboratory on the same host for 20 to 25 generations. To prevent the impact of experience, experimental wasps were dissected as pupa from the host 1 to 2 days prior to hatching to prevent early learning of cues [57].

**Fig. 2:**
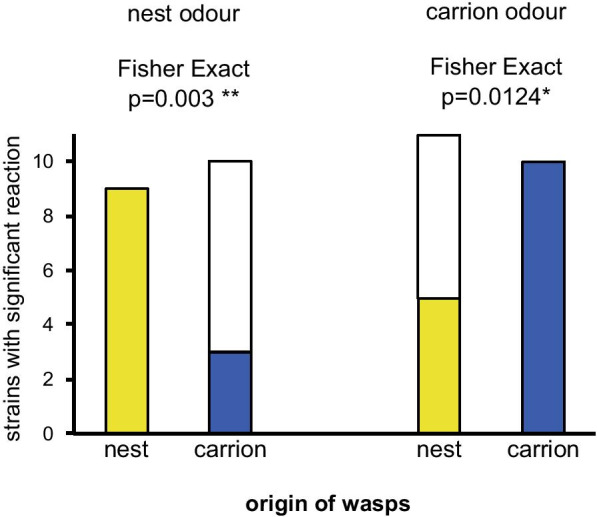
Number of strains of *Nasonia vitripennis* with significant reaction to the odour of bird nests and carrion in olfactometer experiments. Wasp strains originate from bird nests (yellow) or next to carrion (blue). For each strain about 20 wasps were tested. Coloured parts of the bars refer to the number of reacting strains

In olfactometer experiments we studied 11 strains originating from bird nests and 10 strains from carrion. For each strain, about 20 females were tested for their response to nest and carrion odour. Each female was tested only once. The habitat of origin had a strong effect on the reaction of the wasps. More nest strains than carrion strains significantly reacted to nest odour (Fisher’s Exact Test, *p* = 0.003), and more carrion strains than nest strains significantly reacted to carrion odour (Fisher’s Exact Test, *p* = 0.012) (Fig. 2). Statistics for all tested strains are given in Additional file 1: Tables S1 and S2. From the strains which were used in all the further experiments (see below), nest strains N3 and N9 only reacted significantly to odours from bird nests, carrion strains A1, A7, and A19 only reacted to carrion, and nest strain N2 reacted to both (Additional file 1: Table S1).

**Fig. 3:**
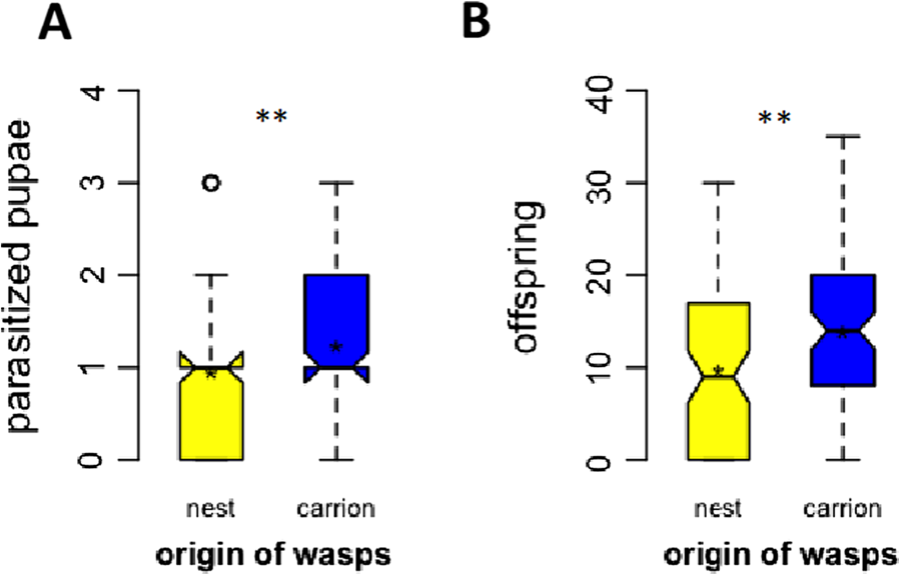
Number of pupae of *Lucilia sericata* carrion flies parasitized by *Nasonia vitripennis* wasps (**A**) and offspring emerging from these pupae (**B**). Wasps were collected in bird nests (yellow, n = 90) or next to carrion (blue, n = 90). The box and whisker plots show minimum, maximum, 1st and 3rd quartile, median and mean as asterisk. **: significant difference at *p* < 0.01 (Mann–Whitney U-test)

Host acceptance was tested with pupae of *L. sericata* carrion flies (Fig. 3), but not with pupae from nest flies. Parasitic nest flies of *Protocalliphora,* the putative main host of *N. vitripennis* in bird nests, are difficult to rear in the laboratory. Wasps from three strains originating from bird nests (N2, N3, N9) and three strains originating from carrion (A1, A7, A19) were tested. Generally, the number of parasitized fly pupae and offspring emerging from the pupae was higher in the three carrion strains compared to the three nest strains (Additional file 2: Figs. S1, S2). Generalized linear models (family “poisson”) followed by ANOVA revealed no difference between strains within each group (nest or carrion). Therefore, data from all nest strains were pooled and compared to the pooled carrion strains using Whitney–Mann U-test, as data were not normally distributed. This revealed highly significant differences between wasps from nests strains and wasps from carrion strains for the number of parasitized fly pupae (Fig. 3; U-test: W = 3189; *p* = 0.008) and the number of offspring emerging from the pupae (U-test: W = 3047; *p* = 0.004).

### Genetic analyses

**Fig. 4:**
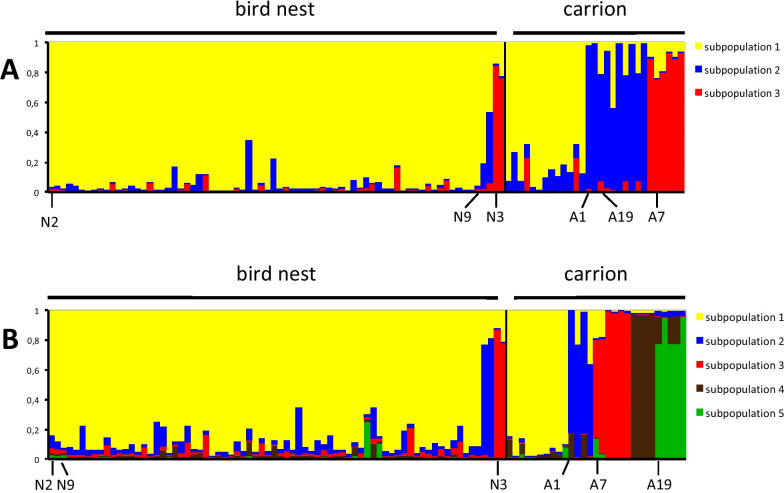
Results from a population genetic analysis using microsatellites of a population of the parasitoid wasp *Nasonia vitripennis* in the park of Hohenheim, Germany, using STRUCTURE v2.3. Wasps were collected in bird nests or next to carrion. In the bar plots each individual is represented by a vertical line coloured according to its probability of assignment to one of either k = 3 (**A**) or k = 5 (**B**) subpopulations. Origin of wasps from bird nests or carrion is indicated above each graph. The affiliation of experimental strains N2, N3 N9, A1, A7, and A19 to subpopulations within the dataset is indicated below each graph

A population genetic analysis using microsatellites was performed using wasps from six laboratory strains (strains from bird nests: N2, N3, N9; strains from carrion: A1, A7, A19; one wasp per strain) as well as other wasps that had emerged from field-collected host baits in bird nests (n = 71) and next to carrion (n = 26) and were not taken into culture. Each single wasp originated from a different host pupa and a different bait. Thus, each analysed female is representative of one female strain. In total, 103 females were studied. The analysis with STRUCTURE v2.3.4 was performed three times. In all three runs there was strong support for either three (k = 3) or five (k = 5) subpopulation clusters (Additional file 2: Fig. S3). The estimated membership coefficients for each individual in each cluster were almost identical in the three runs. Most individuals of subpopulation 1 originated from bird nests, and most individuals of subpopulations 2, 3, 4, and 5 were found next to carrion (Fig. 4A, B). Laboratory strains N2 and N9 were assigned to subpopulation 1, strain A1 to subpopulation 2, strains N3 and A7 to subpopulation 3, and strain A19 to either subpopulation 2 (k = 3) or 5 (k = 5). In subpopulations 1, 2, and 3, between 12 and 30% of the individuals were collected in the alternative habitat and there is no significant difference between subpopulations in the distribution of individuals between main and alternative microhabitat (Fisher Exact test for k = 3: *p* = 1; k = 5: *p* = 0.3298; for data see Additional File 1: Table S3).

Calculations of genetic differentiation revealed considerable inbreeding coefficients (F_IS_, G_IS_) within the individuals representing one female strain each, ranging from 0.318 to 0.413 (Table 1). F_st_-values between subpopulations defined by their origin from bird nests and carrion are always lower than between subpopulations identified by microsatellites. Between subpopulations identified by microsatellites with k = 3, there is low differentiation between subpopulations 1 and 2, as well as 1 and 3, but higher differentiation between 2 and 3. Higher levels of differentiation also exist between most of the subpopulations identified in the k = 5 model. Hedrick’s standardized G′_st_ values also indicate low differentiation between the two microhabitats, but higher differentiation between the subpopulations identified using microsatellites, with highest values ranging up to 0.809. Subpopulation 2 was excluded from the analysis with k = 5 due to the small number of individuals.

**Table 1:** Genetic differentiation among putative subpopulations of *Nasonia vitripennis* in the park of Hohenheim, Stuttgart, Germany

Putative Subpopulations	F_IS_	F_ST_	p	G_IS_	Gʹ_ST_	p
Inbreeding lines within habitat subpopulations	0.318			0.330		
Nest–carrion		0.011	0.065		0.032	0.002**
Inbreeding lines within ms-subpopulations (k = 3)	0.355			0.406		
ms-subpopulations 1–2		0.030	0.002**		0.173	0.002**
ms-subpopulations 1–3		0.120	0.001***		0.536	0.002**
ms-subpopulations 2–3		0.142	0.002**		0.531	0.002**
Inbreeding lines within ms-subpopulations (k = 5)	0.354			0.413		
ms-subpopulations 1–3		0.186	0.003**		0.705	0.002**
ms-subpopulations 1–4		0.261	0.005**		0.809	0.002**
ms-subpopulations 1–5		0.215	0.001***		0.771	0.002**
ms-subpopulations 3–4		0.093	0.001***		0.420	0.002**
ms-subpopulations 3–5		0.064	0.001***		0.349	0.002**
ms-subpopulations 4–5		0.129	0.001***		0.601	0.002**

Subpopulations are based on microhabitats (nest vs. carrion) or on a population analysis with STRUCTURE v2.3.4 using microsatellites (“ms-subpopulations”) [144]. F_IS_, G_IS_, F_ST_ [150–152], Gʹ_ST_ [153, 154] and p-values were calculated using GenAlEx [148, 149]

### Sexual isolation

**Fig. 5:**
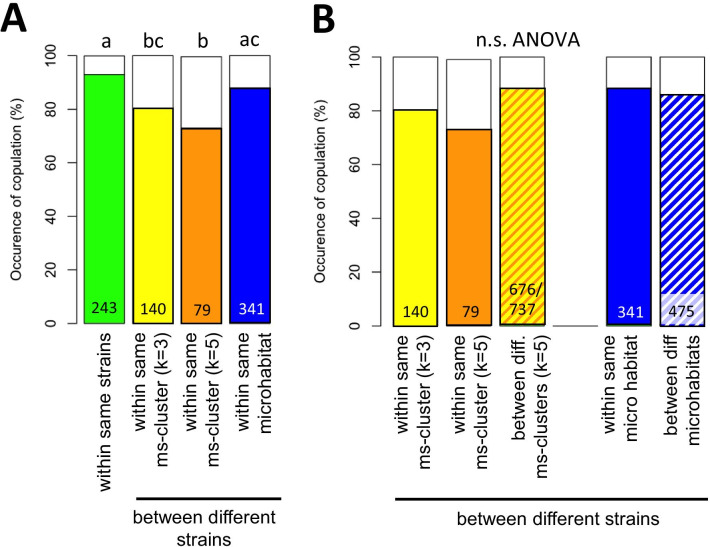
Occurrence of copulation in *N. vitripennis* depending on origin of mating partners. **A** Females and males originate from the same strain (green), or from different strains but the same microsatellite cluster with k = 3 (yellow) or k = 5 (orange) subpopulations, or the same microhabitat (blue). **B** Females and males originate from different strains but the same microsatellite cluster with k = 3 (yellow) or k = 5 (orange), or from different microsatellite clusters (yellow/orange), or originate from the same (blue) or different microhabitats (white/blue). Coloured parts of the bar indicate the percentage of couples with copulation. Numbers in bars indicate numbers of replicates. Different letters above bars in A indicate significant differences at *p* < 0.01 (Tukey-test based on a generalized linear model, family “binomial”). In **B**, ANOVA was based on a generalized linear model, family “binomial”

Sexual isolation was studied with wasps from strains originating from bird nests and carrion (strains from bird nests: N2, N3, N9; strains from carrion: A1, A7, A19). Single pairs of females and males were tested in all possible combinations. Courtship behaviour by males was observed in all experimental couples, in which the male located the female (n = 1059). Thus, there is no isolation due to male mate selection. There was a small, but highly significant decline in the number of females that accepted males for copulations when males were from different strains than females (Fig. 5A; ANOVA based on generalized linear model, family “binomial”: Chi^2^ = 25.00, df = 3, *p* = 0.000). Single comparisons using Tukey-test revealed significant differences between same strain couples and couples from different strains but the same microsatellite cluster, but no differences towards couples from different strains and the same microhabitat. When females and males were from different strains, there were no differences with respect to their origin from the same or from different microsatellite clusters, or from the same or different microhabitats (Fig. 5B; Table 2). Thus, there is a small, significant level of sexual isolation between partners from different strains caused by female mate choice, but no isolation between partners from different microsatellite clusters or different microhabitats.

**Table 2:** Test statistics of experiments on sexual isolation among females and males from different strains in *Nasonia vitripennis*

Response variable	Factors	Chi^2^	df	*p*
Occurrence of copulation	Ms-cluster (k = 3)	0.1269	1	0.722
	Ms-cluster (k = 5)	3.7351	1	0.053
	Microhabitat	1.1870	1	0.276

ANOVA test statistics based on a generalized linear model, family “binomial”, with the occurrence of copulations as response variable and the affiliation to the same or different microsatellite clusters (either based on k = 3 or k = 5 populations) and the microhabitat of wasps (same microhabitat, different microhabitats) as factors

### Postmating isolation

**Fig. 6:**
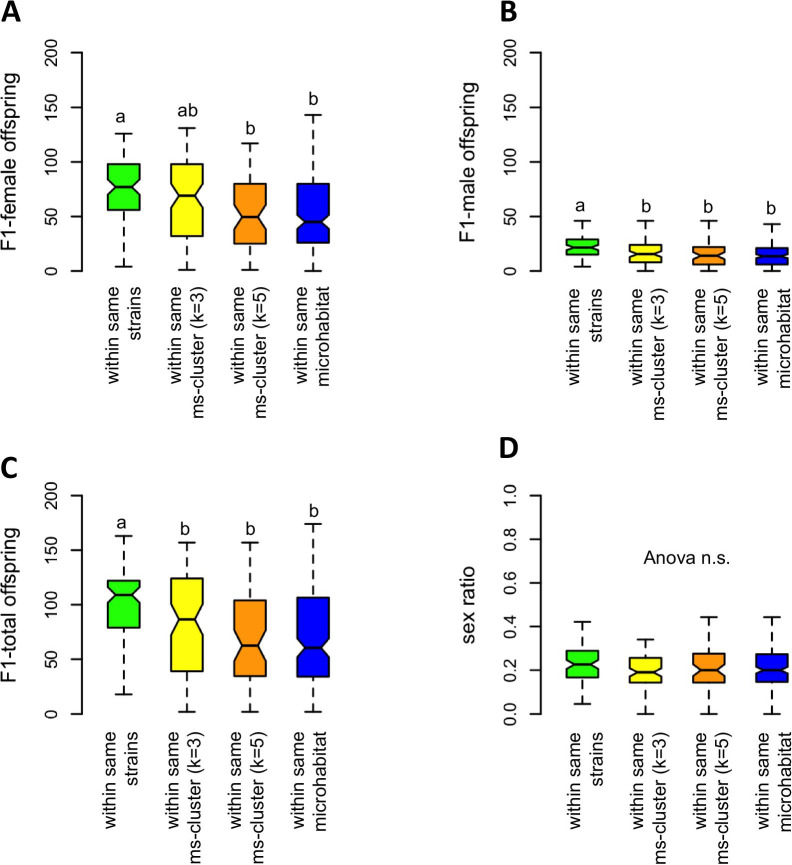
Influence of origin of the mating partners on number and sex ratio of F1-offspring in the parasitoid wasp *Nasonia vitripennis.* **A** F1-female offspring; **B** F1-male offspring; **C** F1-total offspring; **D** sex ratio. Females and males originate from the same strain (green, n = 90), or from different strains but the same microsatellite cluster with k = 3 (yellow, n = 90) or k = 5 (orange, n = 60) subpopulations, or originate from the same microhabitat (blue, n = 180). Box and whisker plots show minimum, maximum, 1st and 3rd quartile, and median. Different lower case letters indicate significant differences (*p* < 0.05, generalized linear models with family “quasipoisson” followed by ANOVA and Tukey-test. For test statistics see Tables 3 and 4

Postmating isolation was studied with wasps from the same strains as above (strains from bird nests: N2, N3, N9; strains from carrion: A1, A7, A19). Females and males were mated in all possible combinations. Only three out of the 540 couples did not produce F1-female offspring. The number of F1-female offspring was significantly influenced by the origin of mating partners from the same strain, from different strains but the same microsatellite cluster with k = 3 or k = 5 subpopulations, or the same microhabitat (Fig. 6A; Table 3). The strain and the habitat of the parental female did not influence the number of F1-females (Table 3). Couples from the same strain had significantly more F1-female offspring than couples from different strains but from the same microsatellite cluster with k = 5 subpopulations, and from the same habitat. There was no difference between couples from the same strains and couples from different strains but from the same microsatellite cluster with k = 3 subpopulations.

**Table 3:** ANOVA test statistics of experiments on the influence of different factors on the number of F1-female offspring as postmating barrier in *Nasonia vitripennis*

Response variable	Factors	Chi^2^	Df	*p*
Number	Origin	23.6315	1	0.000***
F1-female	Female strain	1.6019	1	0.2056
offspring	Female habitat	2.5341	1	0.1114

ANOVA test statistics based on generalized mixed models, family “quasipossion”, with origin (origin of wasps from the same strain, from different strains but the same microsatellite cluster with k = 3 or k = 5 subpopulations, or the same microhabitat), female strain (strains from bird nests: N2, N3, N9; strains from carrion: A1, A7, A19), and female habitat (bird nest or carrion) as factors

To study if the reduction in female offspring is due to the number of eggs laid during the experimental period, due to mortality of the F1-female offspring, or due to partial CI, we also analysed the number of F1-male offspring, the total number of F1-offspring (males and females), and the sex ratio. Like with F1-females, couples from the same strain had significantly more F1-male and F1-total offspring than couples from different strains but from the same microsatellite cluster subpopulations, and from the same habitat (Fig. 6; Table 4). There were no differences in sex ratio between the treatments.

**Table 4:** Test statistics of experiments on postmating isolation between strains in *Nasonia vitripennis*

Response variables	Factor	Chi^2^	Df	*p*
F1-female offspring	Origin of wasps	24.892	3	0.000***
F1-male offspring	“	24.688	3	0.000***
F1-total offspring	“	31.838	3	0.000***
F1 sex ratio	“	3.0007	3	0.3915

ANOVA test statistics based on generalized mixed models, family “quasipossion”, with origin of wasps (from the same strain, from different strains but the same microsatellite cluster with k = 3 or k = 5 subpopulations, or the same microhabitat) as factor

**Fig. 7:**
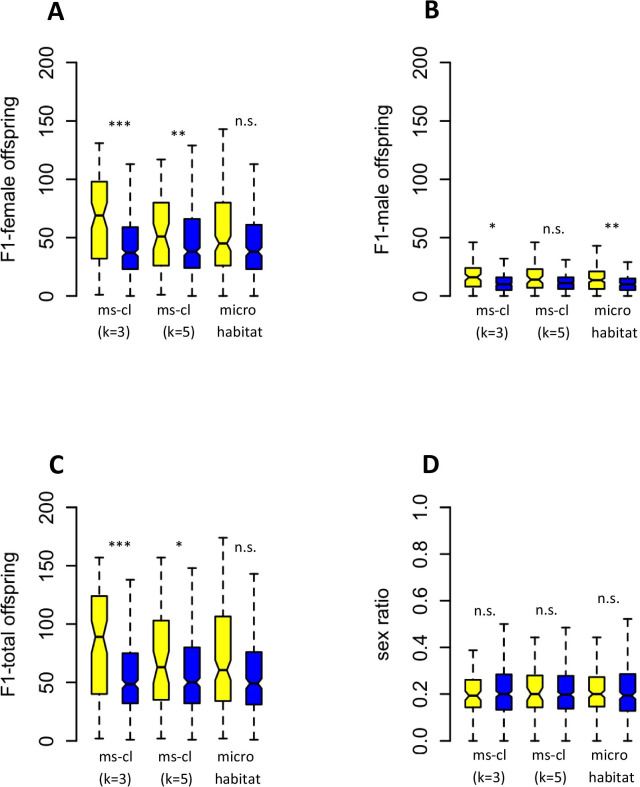
The influence of origin of mating partners on number and sex ratio of F1-offspring in the parasitoid wasp *Nasonia vitripennis.* **A** F1-female offspring; **B** F1-male offspring; **C** F1-total offspring; **D** sex ratio. Mating partners originated from the same or from different microsatellite clusters (with k = 3 or k = 5 subpopulations), or from the same or different microhabitats. Box and whisker plots show minimum, maximum, 1st and 3rd quartile, and median. Levels of significance are based on generalized linear models, family “negative binomial”, followed by Tukey-test. For test statistics see Table 5

To analyse data of couples with females and males from different strains, we used generalized linear mixed model (“negative binomial”) with affiliation to the same or different microsatellite clusters (either based on k = 3 or k = 5 populations), origin of the wasps (same microhabitat, different microhabitats), strain of parental female, and habitat of parental female as factors. This revealed a highly significant impact of affiliation to the same or different microsatellite clusters on the number of F1-female and F1-total offspring, but no significant effect of microhabitat, female strain and female habitat (Table 5; for F1-offspring numbers from each strain combination see Additional file 1: Table S4). In addition, subsequent Tukey-test showed that numbers of F1-female and F1-total offspring in couples from the same microsatellite cluster are significantly higher as compared to couples from different clusters, but that there is no difference in offspring numbers between couples from the same vs. couples from different microhabitats (Fig. 7A, C). The number of F1-male offspring in the treatments with partners from different microsatellite clusters or different microhabitats were always slightly lower (Fig. 7B), and there were significant effects of affiliation to the microsatellite cluster k = 3, of microhabitat, female strain, and female habitat (Fig. 7B, Table 4). The overall mean sex ratio of F1-offspring was 0.23 ± 0.14 (mean ± sd; n = 540) and not influenced by either factor (Fig. 7D, Table 5).

**Table 5:** Test statistics of experiments on postmating isolation between microsatellites clusters and microhabitats in *Nasonia vitripennis*

Response variable	Factors	Chi^2^	Df	*p*
F1-female offspring	Ms-cluster (k = 3)	23.634	1	0.000***
	Ms-cluster (k = 5)	8.905	1	0.003**
	Microhabitat	0.530	1	0.492
	Female strain	0.112	1	0.737
	Female habitat	0.155	1	0.693
F1-male offspring	Ms-cluster (k = 3)	6.099	1	0.0135*
	Ms-cluster (k = 5)	0.052	1	0.820
	Microhabitat	9.212	1	0.002**
	Female strain	33.905	1	0.000***
	Female habitat	34.464	1	0.000***
F1-total offspring	Ms-cluster (k = 3)	23.023	1	0.000***
	Ms-cluster (k = 5)	6.963	1	0.009 **
	Microhabitat	1.782	1	0.185
	Female strain	2.792	1	0.095
	Female habitat	2.576	1	0.108
Sexratio	Ms-cluster (k = 3)	0.426	1	0.551
	Ms-cluster (k = 5)	0.483	1	0.524
	Microhabitat	0.142	1	0.720
	Female strain	1.597	1	0.209
	Female habitat	1.575	1	0.206

ANOVA test statistics based on generalized linear models, family “negative binomial”

### Isolation indices

Isolation indices were calculated using the olfactometer data for ecological isolation, data on the occurrence of copulations for sexual isolation, and data on the number of F1-female offspring for postmating isolation (Table 6). Indices show considerable total isolation between couples from the same strain and couples from different strains but from the same microsatellite cluster or the same microhabitat. This is either based on sexual barriers, postmating barriers, or both. While different microsatellite clusters are separated by postmating barriers, strains from the two different microhabitats are only separated by ecological barriers.

**Table 6:** Isolation indices for different barriers between *Nasonia vitripennis* wasps from different strains but same microhabitat, and wasps from different strains and different microhabitats

Origin of couples	Barrier			
	Ecological	Sexual	Postmating	Total
Same strains vs. diff. strains from the same ms-clusters (k = 3)	0	0.075*	0.054	0.128
Same strains vs. diff. strains from the same ms-clusters (k = 5)	0	0.12*	0.217*	0.328
Same strains vs. diff. strains from the same microhabitat	0	0.033	0.262*	0.259
Diff. strains within the same ms-cluster (k = 3) vs. strains from diff. clusters	0	− 0.048	0.302*	0.258
Diff. strains within the same ms-cluster (k = 5) vs. strains from diff. clusters	0	− 0.093	0.132*	0.039
Diff. strains within the same habitat vs. strains from strains from diff. habitats	0.458*	0.006	0.084	0.525

Data were calculated according to Sobel and Chen [139] using olfactometer data for ecological isolation, data on the occurrence of copulations for sexual isolation, and data on the number of F1-female offspring for postmating isolation. Significant barriers are in bold

## Discussion

To answer the question which reproductive barriers initiate speciation is difficult for two reasons. First, because several barriers can arise during the speciation process [1] study systems must consist of populations that are still exchanging genes at the very early phase of separation [20]. However, most studies on early barriers have used young species pairs (e.g. [103]), allopatric populations [104], or ecologically diverged subpopulations (e.g. [105]). In these systems separation has already proceeded which prevents the identification of the earliest barrier. Second, initial barriers are expected to be only weak at the beginning. Therefore, many experimental replicates are needed for their identification, which requires study organisms that are available in large numbers. Thus, there are only very few studies that allow conclusions on barriers within populations which have not yet diverged [106]. To identify very early barriers of separation in Hymenoptera, we studied ecological isolation, sexual isolation, postmating isolation and population structure within a sympatric population of the parasitoid wasp *N. vitripennis*. Our study revealed three levels of isolation within the population: Between female strains, between subpopulation clusters based on microsatellites, and between ecotypes.

### Ecological isolation

Our study confirms the existence of two different ecotypes in *N. vitripennis*, one occurring in bird nests, and the other in carrion. This has already been suggested previously [107, 108]. However, we could not find any premating or postmating reproductive barriers between these ecotypes except of ecological traits, and the carrion ecotype does not seem to represent a distinct genetic cluster (see below). In contrast, as shown in our microsatellite study, it consists of several, genetically distinct subpopulations.

The ecological differences between the ecotypes refer to their phenology (1), their reaction to host habitat cues (2) and their host acceptance (3). In bird nests, wasps were present from May until end of July after nestlings had left, while they occurred on carrion from May until September. We assume that this difference in phenology is due to the fact that nest wasps are adapted to the presence of pupae of *Protocalliphora* flies which are parasitic on nestlings that occur in nests only until the end of June [109]. In contrast, carrion wasps might be adapted to the longer availability of carrion and pupae from carrion flies. However, we cannot exclude that this finding is an artefact caused by the loss of attractiveness of nest material due to the complete evaporation of the involved volatiles after the second brood has left the nest.

In olfactometer experiments on host habitat location, the reaction of wasps depended on the microhabitat of origin. All strains reacted to the odour of the microhabitat in which they were collected, but only some strains reacted to the odour of the foreign microhabitat. Because wasps were reared for 20 to 25 generations on the same hosts and were dissected before emergence from the pupa to avoid early adult experience [57] this reaction can not have been learned, but must be based either on genetic differences between the strains, or differences in the microbiome [110]. The importance of odours for host habitat location is highlighted by the absence of parasitization in control fly pupae, which were offered without habitat odours in the field. This demonstrates that hosts are only found in those microhabitats, for which an olfactory reaction is present.

In experiments on host acceptance with pupae of the carrion associated fly species *L. sericata* [111], the number of parasitized pupae and the number of emerging total offspring was significantly higher in wasps from carrion as compared to wasps from bird nests. Again, the higher acceptance of carrion fly pupae by carrion wasps must be inherited, because all experimental wasps were reared on the same hosts for many generations and dissected in the pupal stage from the host. It remains to be examined if the reduced offspring numbers in nest wasps is due to reduced parasitization (host acceptance), or because of a higher mortality during development, i.e. an inferior host suitability of carrion flies. Although technical reasons prevented us from conducting the reciprocal experiment with *Protocalliphora*, which is the main host of wasps from bird nests, these data clearly demonstrate a better adaptation of wasps collected from carrion to pupae of carrion flies. However, it might well be that the level of ecological segregation is underestimated due to the lack of experiments with *Protocalliphora*.

Based on these and other traits, the ecotypes meet many criteria for host races of plant feeding insects [14, 112, 113]: They are associated with different habitats and hosts which is demonstrated by their specific reaction to habitat odours, their host acceptance and possibly their phenology. They display a correlation between host choice and mate choice due to their inbreeding behaviour between siblings, at least one (the carrion type) has a higher fitness on its specific hosts, and they occur in sympatry. In contrast to host races, however, only one of the ecotypes is genetically differentiated.

### Sexual isolation

While males always performed courtship behaviour regardless of the origin of the females, females were less likely to accept males for copulation when they originated from different strains but the same microsatellite cluster. In contrast, there were no significant differences in the number of copulations between strains from the same or from different microsatellite clusters, and between the same or different microhabitats. Thus, there is slight sexual isolation between inbreeding strains caused by mate choice decisions from the females, but no isolation between microsatellite clusters or microhabitats. This mate choice decision by the female is most likely based on the male mandibular pheromone which is applied on the female antennae by the males during courtship [114]. It must have a genetic basis and can not have been induced by the developmental experience of the females as described for the related *L. distinguendus* [58], because all wasps were reared under identical conditions and dissected as pupa from the host prior to hatching.

### Postmating isolation

Couples consisting of females and males from the same strain had more F1-female offspring as compared to couples from different strains but from at least one of the identified microsatellite cluster or the same microhabitat. Likewise, females produced more F1-female offspring after copulations with males from the same microsatellite cluster as compared to copulations with males from a foreign cluster. The origin of mating partners from the same or from a different microhabitat had no effect. Obviously, postmating reproductive barriers exist between inbreeding female strains, and between microsatellite clusters, but not between ecotypes.

Generally, the reduction in F1-female offspring in couples with partners from different female strains and microsatellite clusters can have different reasons: (1) Genetic incompatibilities according to the Dobzhansky–Muller (D–M) model due to nuclear–nuclear or nuclear–mitochondrial interactions, which are both described for *Nasonia* [59, 62, 79]. This would result in a reduction of the total number of F1-offspring due to higher mortality of hybrid F1-females during development, and consequently a change in the normal, female biased sex ratio [49] towards more male offspring. (2) Total or partial cytoplasmic incompatibility (CI) induced by *Wolbachia* or other endosymbionts causing mortality of the hybrid female zygote (female mortality type), or its conversion to a male (male development type) [86]. All types of CI would lead to changes in sex ratio. Female mortality CI would also result in a reduced total number of F1-offspring similar to D–M incompatibilities. Male development CI would result in an increased number of male offspring and no change in the total number of F1-offspring. (3) A lower reproductive investment by females after matings with males from a different strain. (4) A noncompetitive postmating, prezygotic barrier (PMPZ) acting between copulation and fertilization [1]. Because we observed no differences in sex ratio in all experiments on postmating isolation, D–M incompatibilities and CI of any type is unlikely as explanation. Male development CI can also be excluded because the number of F1-male offspring did not increase and the total number of F1-offspring declined. A lower investment by females is also unlikely as reason because females mate only once and should optimize their own offspring numbers, regardless of the quality of their mates. Therefore we favour PMPZ as explanation. PMPZ involves the interaction of gametes and/or reproductive tissue or reproductive proteins and can result in several barriers like reduced sperm transfer, inefficient sperm storage, or the failure of sperm to fertilize eggs [115]. We assume that PMPZ has reduced the amount of sperm available to females for fertilization of eggs. Therefore female laid fewer F1-female eggs and also less male eggs to maintain an optimal sex ratio to avoid local-mate competition [48, 49]. Interestingly, PMPZ has been shown to evolve very fast [116], and to occur as first or only barrier between closely related taxa or populations of *Drosophila* [115, 117]. We are currently studying this hypothesis as well as further barriers like sexual isolation, and intrinsic postzygotic barriers like inviability and fecundity of hybrids that could also contribute to isolation between strains.

While our data demonstrate a reduction of F1-female offspring after outbreeding, we could not find an indication for inbreeding depression, i.e. a decrease in fitness after mating with siblings. This is remarkable. Generally, inbreeding is assumed to result in higher levels of homozygosity and consequently inbreeding depression [118]. This effect seems to be lower in haplodiploid taxa like Hymenoptera, mites [119], and ambrosia beetles [42] than in diploid species which is explained by prolonged inbreeding and purging of the genetic load by the regular exposition of deleterious mutations to selection in haploid males [42]. Outbreeding, on the other hand, is generally associated with elevated fitness compared to inbreeding. However, above certain levels of parental dissimilarity, outbreeding depression can occur in hybrids, by genetic intrinsic incompatibility due to underdominance, chromosomal rearrangements or deleterious epistatic interactions, or extrinsic postzygotic barriers like maladaptation of hybrids to paternal habitats [120, 121]. While outbreeding depression is well studied in plants, there are much less studies on animals so far. Interestingly, most animal species with outbreeding depression have a similar biology to *N. vitripennis*. They either perform selfing as equivalent to inbreeding like freshwater snails [122], or nematodes [123], or they are haplodiploid like mites [124], or do both like in ambrosia beetles [42]. Possibly, inbreeding or selfing as well as haplodiploidy are a prerequisite to outbreeding depression.

### Genetic population structure based on microsatellites

The analyses of microsatellite markers indicate a remarkable level of inbreeding within the female lines, and the existence of one subpopulation cluster, which is largely restricted to bird nests, and two or four clusters largely restricted to carrion as microhabitat. While a high level of within patch inbreeding was also found in a Swedish population of *N.* *vitripennis* [125], the existence of subpopulation clusters within a local population has not been reported before for *N. vitripennis*. In support of the identified population structure, F_st_ and G_st_-values showed stronger differentiation between the microsatellite subpopulations but only low genetic differentiation between wasps from the two microhabitats. Remarkably, F_st_ and G_st_-values between microsatellite subpopulations are within the range or even higher than values reported by [96] for geographically distant populations of *N. vitripennis* within North America or Europe, and even between North America and Europe. In addition, our mating experiments revealed significantly lower F1-offspring numbers in couples from different microsatellite subpopulations as compared to couples from the same subpopulation, while this was not the case for couples within and between different microhabitats. Obviously, microsatellite subpopulations are not only genetically different, but also separated by the same postmating barrier that is present between the different female strains.

### The population structure of *N. vitripennis* and its emergence

Taken together, the data demonstrate that our local population of *N. vitripennis* is structured on three levels: Between inbreeding strains, between genetic clusters based on microsatellite analysis, and between ecotypes. Strains are characterized by considerable inbreeding coefficients, and are separated by slight sexual isolation and postmating isolation from other strains of the same microsatellite cluster and from other strains of the same ecotype. Strains from different microsatellite clusters are also genetically distinct and separated by postmating isolation. In total, barriers between inbreeding strains lead to a total isolation ranging from 0.128 and 0.328, and between microsatellite clusters from almost zero (0.039) to 0.258 (Table 6). In contrast, the ecotypes of *N. vitripennis* are not separated by sexual isolation or postmating barriers, but only by considerable ecological barriers leading to a total isolation of 0.525 (Table 6).

Remarkably, these ecotypes consist of different microsatellite subpopulations. Subpopulation 1, which is the largest, was mostly collected in bird nests and probably represents the ecotype that is adapted to bird nests as host microhabitat. Accordingly, laboratory wasp strains N2 and N9 from subpopulation 1 showed a significant reaction to odours from bird nests in the olfactometer experiments, and a lower parasitization of fly pupae from carrion flies in experiments on host acceptance. In contrast, subpopulations 2, 3, 4, and 5 were mostly collected next to carrion and are probably adapted to this microhabitat, forming the carrion ecotypes. Therefore, A1, A7, and A19 belonging to subpopulations 2 and 3 (k = 3), or 2, 3, and 5 (k = 5), only reacted to carrion and had a higher acceptance of carrion fly pupae. However, the separation between the two ecotypes and microhabitats is not strict and between 12 and 30% of the individuals of strains 1, 2, and 3 were also collected in the alternative habitat. For instance, N3 was genetically assigned to the carrion subpopulation 3, but was collected in bird nests and only reacted to nest odours. Possibly, this strain is intermediate between the two ecotypes.

The emergence of premating and postmating reproductive barriers between inbreeding strains as first barrier in our population is consistent with Askew’s inbreeding hypothesis [39], stating that inbreeding could lead to reproductive isolation by genetic drift [40, 41]. Alternatively, postmating barriers could also be explained by CI due to qualitative or quantitative differences in the presence of CI-inducing bacteria like *Wolbachia*, and premating barriers could result from differences in developmental experience influencing mate choice decisions of the females [58]. However, the influence of CI can be excluded by our data on F1-offspring and F1-sex ration, and the role of experience due to our experimental procedure (see above). Therefore, at present we believe that Askew’s inbreeding hypothesis is the most likely explanation. As mentioned above, mating in *N. vitripennis* occurs mostly between female and male offspring from the same maternal female directly after emergence from the host [97], or sometimes even within the host [94]. This could lead to reproductive isolation and promote separation. Remarkably, other animal species with outbreeding depression also perform inbreeding [42] or selfing [122].

**Fig. 8:**
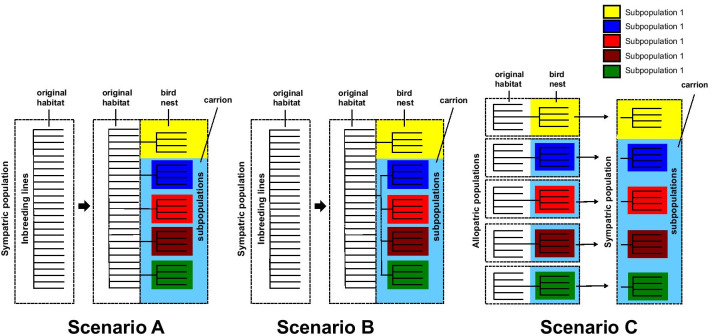
Hypothetical scenarios for the emergence of the observed pattern of subpopulations and ecotypes of *Nasonia vitripennis*. **Scenario A** Inbreeding lines evolve into subpopulations in sympatry and become independently adapted to their respective microhabitats. **Scenario B** Inbreeding lines evolve into two subpopulations in sympatry. One subpopulation becomes adapted to bird nests as microhabitat and one subpopulation becomes adapted to carrion. Subsequently, the carrion subpopulations splits up into 2 or 4 separate subpopulations. **Scenario C** Subpopulations became adapted to their respective microhabitats in allopatry and become sympatric by immigration into the study area

With respect to the evolution of the microsatellite subpopulations and the resulting ecotypes three scenarios are conceivable. Thereby it is unclear if the initial population consisted of bird nest specialists, from which carrion specialists evolved by host switching, or vice-versa, or if it consisted of generalists, that evolved into microhabitat specialists for bird nests and carrion. The fact that all other species of the genus *Nasonia* occur in bird nests [126] could indicate that *N. vitripennis* has originated from a bird nest specialist. However, its larger host range comprising pupae of blowflies, fleshflies, houseflies, and others [126] suggests that it is a generalist. In any case, microsatellite subpopulations could have evolved in sympatry from the inbreeding lines and became adapted to their microhabitat, either once for the bird nest ecotype and several times independently for the carrion ecotype (Fig. 8, scenario A), or only once for each ecotype (scenario B). Thereby the adaptation to the microhabitats could be based on new mutations, or on ancestral alleles present in low frequencies in the original population [127]. However, the observed pattern could also be the result of immigration of ecotypes that evolved their adaptations in allopatry (Fig. 8, scenario C). This agrees with the idea that ecological speciation starts in allopatry and continues in sympatry [128]. Note that scenarios 1 and 3 assume that phenotypes evolved repeatedly in genetically separated clusters [129], i.e. the reaction of wasps from strains A1, A7, and A19 to odours from carrion, and their higher acceptance of carrion fly pupae would have evolved independently. To identify the most likely of these hypothetical scenarios, an analysis of the ecology and the population genetics of *N. vitripennis* populations from a larger geographic area, e.g. Central Europe, is required.

While it is unclear if the two different ecotypes evolved in sympatry, our results clearly demonstrate that they are able to co-exist in sympatry. This supports the idea of sympatric speciation also for parasitoid wasps, which was only shown for the braconid species *Diachasma alloeum, Utetes canaliculatus*, and *Diachasmimorpha mellea* that have separated in sympatry as a consequence of the divergence of their *Rhagoletis* hosts [53, 55]. If true, the putative separation in *N. vitripennis* was not induced by host radiation as in these braconids, but occurred independently due to the presence of two different host-microhabitats. It would therefore represent the only parasitoid example demonstrating that incipient sympatric host race or ecotype formation is possible even in the absence of sympatric host radiation.

## Conclusions

Our study sheds light on the earliest steps of ecological separation in a hymenopterous parasitoid. In contrast to the assumption that separation by natural divergent selection generally starts with ecological barriers [130, 131], we found sexual isolation and postmating isolation as first barrier between inbreeding strains that are not separated ecologically. This points to inbreeding as a hitherto underestimated mechanism in evolution and speciation [39, 42, 132–134]. In addition, the two ecotypes of *N. vitripennis*, one occurring in bird nests and the other on carrion, do not form two, but several genetically distinct, sympatric subpopulations. This indicates the complexity of ecotype formation. To further substantiate the hypothesis that this separation occurred in sympatry, the ecology and the population structure of *N. vitripennis* populations from Central Europe is required. Finally, the study highlights the general need for more studies within seemingly homogenous, sympatric populations for the identification of the earliest barriers in the speciation process [20]. Because barriers at the onset of separation are very weak per definition, a large number of replicates are required to obtain sufficient statistical power for the identification of these barriers. Unfortunately, this is very difficult to realize in most study systems.

## Methods

### Field experiments

To study phenology and to establish laboratory cultures of *N. vitripennis*, wasps were collected using bait bags with fly pupae in the park of the University of Hohenheim (Stuttgart, Germany). The park has 35 ha of meadows, trees, understory, and ponds and is very species-rich. A 24-h survey in 2013 (“Geo-Tag der Artenvielfalt”) revealed the presence of at least 365 plant species and 846 animal species in the area. *N. vitripennis* was collected from the end of March to the end of October 2012 in bird nests and next to carrion. Bait bags were made of aluminium gauze (4.5 cm × 1 cm, 1.5 mm mesh width) and contained five fly pupae of the green blowfly *Lucilia sericata* (Meigen 1826) (Diptera: Calliphoridae). Fly pupae were obtained from a pet store as larvae, allowed to pupate, and stored at 4 °C until being used. Single bait bags were placed below the nesting material in 29 bird boxes (Schwegler, Germany) situated up to 3.5 m high on trees. The boxes contained nests of tits (*Parus major* L., *Cyanistes caeruleus* L., *Parus palustris* L.) from the current breeding season. One bait bag was also placed next to each of 15 steel grid cages (215 × 115 × 55 mm) containing fresh carrion of small rodents (*Mus musculus* L*., Microtus arvalis* (Pallas 1778), *Meriones unguiculatus* (Milne-Edwards 1867)). Eight control baits without nest boxes or carrion were attached to twigs at different locations in the park. After 1 week, fly pupae from the baits were replaced by new fly pupae, taken to the laboratory and incubated at 25 ± 1 °C and 60% r.h. in a thermostatic cabinet (Aqualytic^®^ AL-185-4). These conditions were used for all cultures. Emerging wasps were determined under a microscope using [135].

### Experimental wasps

To establish inbreeding strains for laboratory cultures, wasps emerging from single host pupae from the bait bags were placed in Petri dishes with *L. sericata* pupae (approx. 6 g) that had been killed by freezing at − 20 °C for a minimum of 1–2 days. By using only wasps from one single host pupa for each strain, we simulated the natural situation in which offspring emerging from one host are often inbreeding [97], but still maintains at least some genetic diversity. Note that this approach is different from generating artificial isofemale lines, which are used to produce genetically homogenous individuals to study the genetics of certain traits (e.g. [136]). Strains from nest boxes and from carrion were kept in different rooms at constant light. Cultures were provided with dead host pupae three times a week. For most experiments, except for the reaction to host habitat odours, we selected three nest strains (N2, N3, N9) and three carrion strains (A1, A7, A19) from the laboratory cultures. These strains were collected in different collection weeks and with different baits. To prevent the impact of experience, all strains had been reared under identical conditions on dead *L. sericata* pupae for many generations (see below). In addition, experimental wasps were dissected as pupa from the host pupae 1 to 2 days prior to hatching. Therefore, all identified differences between strains must be genetically fixed and can not be attributed to learning behaviour [57].

### Reaction to host habitat odours in the olfactometer

In a four-chamber olfactometer we tested for the presence of a positive reaction to the host habitat odour of bird nests in nine nest and 10 carrion strains, and for the presence of a positive reaction to carrion odour in 11 nest strains and 10 carrion strains (Additional file 1: Table S1). Up to the experiments, the strains had been reared for 20 to 25 generations after being collected. Experimental wasps were dissected from the host to exclude experience as described above and kept in groups of 10–20 individuals in perforated Eppendorf tubes prior to the experiments. Each wasp was used only once. We tested the reaction of about 20 female wasps for each strain. Odour samples of nest material had been taken from a nest box in which parasitization of fly pupae by *N. vitripennis* was recorded. Odour samples from carrion originated from a dead mouse that was exposed in the field for 2 weeks in June/July and was infested by microorganisms, feeding maggots of *L. sericata*, and other arthropods. Until being used, the nest material and the mouse cadaver were stored in odour-impermeable roasting bags at 4 °C (nest material), or at − 23 °C (carrion). To obtain standardized odour samples, 3.5 g nest and carrion material was tested for its attractiveness to females from nest strain N29 and carrion strain A7, respectively. Odour extracts were produced by extracting 3.5 g attractive material in a 100 ml bottle (Duran^®^) together with 20 ml dichloromethane at room temperature. After 24 h the extract was filtered and stored in in glass vials (22 ml, Supelco^®^) at − 23 °C.

**Fig. 9:**
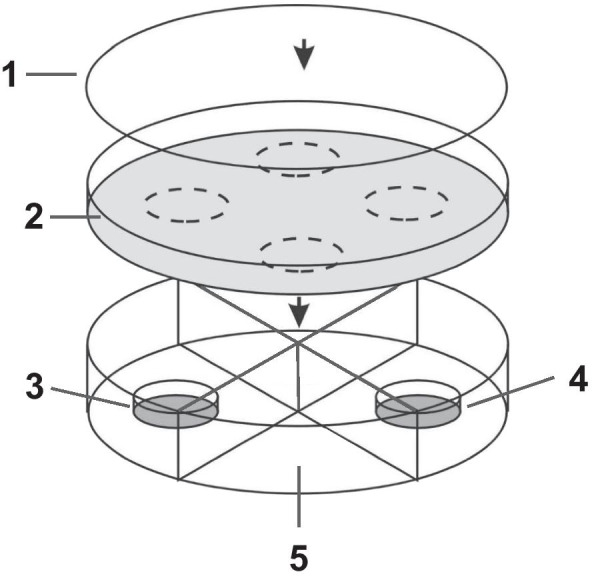
Static 4-chamber olfactometer to test the reaction of female *N. vitripennis* to odours of bird nest and carrion. 1: glass plate; 2: walking arena; 3: test field; 4: control field; 5: transition zone

The static 4-chamber olfactometer according to [137] consists of a polyvinyl chloride cylinder, which is divided into four chambers (Fig. 9). The cylinder is covered with metal gauze (mesh size 0.2 mm) as walking arena on which the wasps can move freely above the four chambers. Every chamber contains a glass dish (Ø 40 mm, height 7 mm). In one chamber the dish contained a filter paper treated with host habitat odour extract (test), the dish in the opposite chamber contained a filter paper treated with dichloromethane (control). The intermediate chambers served as transition zones and remained empty.

Individual wasps were separated into Eppendorf tubes 15 min prior to the experiments. For the experiment, the Eppendorf tube with the wasp was opened and placed in the centre of the arena, which was covered with a glass plate (thickness 2 mm). As soon as the wasp entered the arena, the behaviour (walking, sitting, other) and the location of the wasp (above the test, control, or the two transition chambers) was recorded for 5 min using the software “The Observer^®^ 5.0” (Noldus, 2003, Wageningen, NL). A positive reaction to a host habitat odour was recorded for those strains where allocation times above the test chamber were significantly longer than times above the opposite control chamber. Females which spent < 150 s stationary were considered inactive and excluded from the analysis. After each experiment, the top and bottom of the arena, as well as the bottom of the glass plate were cleaned with 70% ethanol and the olfactometer was rotated by 90°. A maximum of five females from each strain were tested per day. To account for daily response variation due to weather [138], positive controls were performed on each experimental day with females from strain N29 for nest odour and A7 for carrion odour. When these wasps failed to respond, all data from that day were discarded.

### Host acceptance of pupae of carrion flies

Experiments were performed with six strains (N2, N3, N9, A1, A7, A19) from the laboratory culture. After being collected, wasp strains were reared on identical host species (dead *L. sericata* pupae) for 80 to 100 generations. As described above, wasp pupae were dissected from the host pupae and placed into Eppendorf tubes with perforated lids in groups of 10 female and four male pupae for eclosion and subsequent mating. Adult wasps were transferred into Petri dishes (ø 55 mm) and allowed to feed for 24 h on sucrose solution (10%) on filter paper. Thus females had the opportunity to mate and to take up energy for egg maturation. For experiments, single females were placed in Petri dishes (ø 55 mm) with five pupae of *L. sericata* each. After 6 h, wasps were removed and pupae were kept until emergence. The numbers of fly pupae that had been parasitized by each female and the offspring of each female were counted after 3 weeks. From each strain, 30 wasps were tested.

### Sexual isolation

To study sexual isolation, mating experiments were performed with females from three strains originating from bird nests (N2, N3, N9) and three strains originating from carrion (A1, A7, A19) in all possible combinations. After being collected wasp strains were reared on identical host species (dead *L. sericata* pupae) for 60 to 75 generations. To obtain virgin insects for the experiments, wasp pupae where dissected from host pupae as described above, sexed and kept separated by sex in groups of up to 10 individuals in Eppendorf tubes. Experiments were performed in arenas consisting of the bottom of a Petri dish (ø 55 mm) covered with a glass plate. The arena was placed under a digital camcorder (TK-1480BE Color Video Camera, JVC, Yokohama, Japan). Single virgin females (max. age 3 d) were placed in the arena and allowed to calm down for several minutes before one virgin male was added. The observation was finished when copulation was observed or after 10 min. Per combination between 20 and 52 couples were studied.

### Postmating isolation

Experiments were performed with females from three strains from bird nests (N2, N3, N9) and three strains from carrion (A1, A7, A19), which were reared on dead *L. sericata* pupae for 20 to 100 generations. For copulation, single female and male wasps from all strains in all combinations were placed into small chambers (40 × 20 × 15 mm) made of acrylic glass closed with a transparent lid. After copulation was observed the wasps were left in the chamber until the next day. Then, single females were transferred into Petri dishes (60 × 15 mm) with 10 pupae of *L. sericata* for parasitization and kept there until their death. No additional food was provided. Host pupae had been killed by freezing at − 20 °C and subsequently thawed at room temperature (see above) to rule out potential differences between the strains based on any ability to suppress the host immune defence. Care was taken to make sure that the host puparia were all of the same size and quality. After 4 weeks the offspring was sexed and counted.

### Calculation of isolation indices

Reproductive isolation (RI) is the strength of any reproductive barrier and represents an estimate how much gene flow is reduced by this barrier [1]. Based on Sobel and Chen [139] RI varies from − 1 (the barrier allows for gene flow only between heterospecifics) over 0 (gene flow is random) to 1 (only gene flow between conspecifics). RI was calculated according to the following formula:

$$1-2*\frac{(H)}{\left(H\right)+(C)}$$

H refers to events, which enable gene flow between different inbreeding lines, microsatellite clusters or ecotypes (i.e. frequency of mating or the number of offspring after mating), while C refers to events, which enable gene flow between different inbreeding lines, microsatellite clusters or ecotypes. RI for ecological isolation was calculated based on the data from olfactometer experiments. To calculate RI for sexual isolation, we used the data of the mating experiments presented here. To calculate RI for postmating isolation we used the mean number of female F_1_ offspring.

Total isolation (T) varies between 0 (no isolation) and 1 (full isolation between populations). It was calculated as the sum of reproductive isolation caused by all different barriers during the life history of the wasps, starting with the premating barriers ecological isolation, and sexual isolation, and ending up with postmating isolation, i.e. the number of F1 ♀ offspring. For each barrier, we used the absolute contribution (AC). It considers this part of gene flow that has not already been prevented by previous stages of reproductive isolation [140]. It was calculated as follows:

AC for the first barrier: AC_1_ = RI_1_.

AC for the second barrier: AC_2_ = RI_2_ * (1 − AC_1_).

AC for the third barrier: AC_3_ = RI_3_ * [1 − (AC_1_ + AC_2_)].

Total isolation (T) was calculated using the following formula:

$$T=\sum_{i=1}^{m}ACi$$

### Population genetic analysis

A population genetic analysis was performed with 103 females to study the population substructure. We used one individual wasp from each of the six laboratory strains N2, N3, N9, A1, A7, and A19 as well as 96 wasps (71 from bird nests and 26 from carrion) that had emerged from field-exposed host baits with alive fly pupae (see above) that were not used for further cultures. From these, one female per bait was analysed, each representing one female strain. Females were extracted using the DNeasy Blood & Tissue Kit (Qiagen^®^, Venlo, NL) following the manufacturer’s instructions. The tissue was mechanically ground and proteinase K enzyme was added. After incubation at 56 °C, the solution was washed in repeated steps with buffer solutions and subsequent centrifuging. All PCRs were carried out in TGradient^®^ 96 (Biometra, Göttingen, GER) and Techne PrimeG^®^ (Cole-Parmer, Stone, UK) thermocycling machines.

Eight microsatellite markers were selected based on [141]. Loci on all five chromosomes of *N. vitripennis* were chosen to achieve a better coverage of the genome and to account for different selection strengths between chromosomes (Table 7). The Qiagen^®^ Multiplex PCR Kit (Qiagen, Venlo, NL) was used for amplification of microsatellite loci in 96-well plates with a volume of 12.5 µl per reaction (6.25 µl Qiagen^®^ Multiplex PCR Master Mix 2×, 2.5 µl Qiagen^®^ Q-Solution, 1.25 µl primer mix (10 mM) and 1.5 µl RNAse-free water). The primer mix contained two primer pairs with different fluorescent markers (HEX: Hexachloro-Fluorescein, 6FAM: 6-Carboxyfluoresceine, Applied Biosystems, Waltham, MA, US). All sequences were amplified using the same PCR profile. The reaction was initiated through incubation at 95 °C for 15 min, followed by 25 cycles at 94 °C for 30 s, 60 °C for 90 s and extension at 72 °C for 60 s, with a final elongation at 72 °C for 45 min and cool-down to 2 °C until further usage. Fragment length analysis was carried out by Eurofins Genomics (Ebersberg, Germany) using the ROX500 size standard and an ABI-D filter system on a ABI 3130 XL sequencing machine (Applied Biosystems, Waltham, MA, US). Raw fragment length data were examined and the quality of individual signals/peaks checked with PeakScanner v1.0 (Applied Biosystems^®^, Waltham, MA, US) according to [142]. The software MICROCHECKER v2.2.3 [143] was used to check the data set for false allele sizes as well as existence of null-alleles or allelic dropout.

**Table 7:** Markers used in the microsatellite analysis

Marker	Chromosome	Position (cM)	No. alleles	Sequence (5ʹ → 3ʹ)
Nv109	5	42.7	20	GCTTACTCTCGGGAACTGGA CGAGCATTAACCATCAGCAG
Nv114	4	48.6	19	ATGGGCAATAAAACGAAACG CATCCTTGCGGAGACACTAA
Nv209	2	39.8	13	CCAACTTCTTATTCGTAAGGGAA ACCATTCGCTGGCTGGTA
Nv217	3	97.4	23	AATGGCATTATGCGAATGA CTGCTCTCTGCATGAATCTTT
Nv306	2	48.1	6	TGCTCGGATTTCGAACATTT GCGGATGTTGTTCCGTTATT
Nv311	1	50.3	17	ACTGGCGAAAGCTCAAACC TCGAGCTTTGTTCTGGGATA
Nv319	3	12.6	24	TTTGAGGTTATGCGTCGTTTC GAGCGGAGTGCTTCATTCAG
Nv321	4	89.3	9	CGGTGAGACTCGTGAGATGA AACCGCAGCTCTCAACATTT
		Average	16.37	

Analyses were performed using the software STRUCTURE v2.3.4 [144] and the online tool CLUMPAK [145]. We tested a *k*-range of 1–12, each *k* with 30 iterations. Running length was 1.000.000 MCMC replicates with a burn-in of 100.000 using the admixture model with correlated allele frequencies [146]. As sampling location information [147] we included the origin of the single wasps from bird nests or carrion. The structure analysis was repeated three times to check for consistency of results. Genetic differentiation among populations was quantified using GenAlEx 6.502 [148, 149]. Following earlier microsatellite studies with *N. vitripennis* [96, 97] we calculated pairwise F_ST_ [150–152], Hedrick’s Gʹ_ST_ [153, 154], and p-values following G-statistics (as implemented in GenAlEx 6.502) with 999 permutations.

### Statistical analyses

Statistical analyses were carried out using R [155]. Numeric data from olfactometer experiments (Additional file 1: Tables S1, S2), i.e. the allocation time above the test, control, or the two transition chambers were analysed using linear mixed models [156], or generalized linear mixed models [157] depending on distribution and homogeneity of variances. Multiple comparisons were performed with post-hoc Tukey’s test [158]. The number of strains from each of the habitats that significantly reacted to one of the two odours in the olfactometer experiments, and the distribution of individuals from the subpopulations between main and alternative microhabitat were analysed using Fisher Exact Test (Fig. 2). As numeric data from experiments onhost acceptance, i.e. numbers of parasitized pupae and offspring did not have a normal distribution, the differences between strains were analysed using generalized linear models (family “poisson”) [157]. Because this revealed no difference between strains within each group (nest or carrion), data between nest and carrion strains were compared using non-parametric Mann–Whitney U-test. The occurrence of copulations were compared using generalized linear models, family “binomial”, [157], followed by ANOVA and Tukey-tests for single comparisons [158]. F1-female offspring numbers from experiments on postmating barriers were compared by using generalized linear models, “quasipoisson” and “negative binomial”, followed by ANOVA and post-hoc Tukey’s tests [158].

## Supplementary Information

**Additional file 1: Table S1.** Test statistics from olfactometer experiments with strains of *Lariophagus distinguendus* from bird nests and carrion tested on the odour of bird nests or carrion. The odour field contained samples of bird nests or carrion. Strain abbreviations with “N” refer to strains collected in bird nests, strains with “A” were collected next to carrions. Different letters within the same row indicate significant differences in allocation time between fields (p < 0.005). Single comparisons were made using Tukey-test based on linear mixed models or generalized mixed models with field as factor and observation as random factor (Table S2, separate file). Lines with significant differences between odour field and control field 2 are shaded. **Table S2.** Test statistics from olfactometer experiments with strains of *Nasonia vitripennis* from bird nests and carrion tested on the odour of bird nests or carrion. Strain abbreviations with “N” refer to strains collected in bird nests, strains with “A” were collected next to carrions. **Table S3.** Distribution of individuals between main and alternative microhabitat in the different subpopulations identified by microsatellites for k = 3 and k = 5 subpopulations. The main habitats are bird nests for subpopulation 1 and carrion for subpopulations 2–5. **Table S4.** Mean number (± S.D.) of F1-female offspring from couples consisting of females and males from different strains. Data from intra-strain couples are shaded.

**Additional file 2: Fig. S1.** Box and whisker plot of pupae of *Lucilia sericata* carrion flies parasitized by *Nasonia vitripennis* wasps. N2, N3, N9: Wasp strains that were collected in bird nests (yellow, n = 30 per strain). A1, A7, A19 Wasp strains that were collected in next to carrion (blue, n = 30 per strain). The plots show minimum, maximum, 1st and 3rd quartile, median, outliers as circles and mean as asterisk. **Fig. S2.** Box and whisker plot of offspring emerging from pupae of *Lucilia sericata* carrion flies parasitized by *Nasonia vitripennis* wasps. N2, N3, N9: Wasp strains that were collected in bird nests (yellow, n = 30 per strain). A1, A7, A19 Wasp strains that were collected in next to carrion (blue, n = 30 per strain). The plots show minimum, maximum, 1st and 3rd quartile, median, outliers as circles and mean as asterisk. **Fig. S3.** Putative population structure of the parasitoid *N. vitripennis* in the Hohenheim Park (Germany) based on Delta K. Results from three microsatellite analyses (Run 1-Run 3) using STRUCTURE v2.3.4 [159], and the online tool CLUMPAK.

## Data Availability

The datasets supporting the conclusions of this article will be made available in 10.5061/dryad.8kprr4xn3.
