# Supplementary material for: The emergence of ecotypes in a parasitoid wasp: a case of incipient sympatric speciation in Hymenoptera?
Source: BMC Ecol Evol. 2021 Nov 15;21:204. doi: 10.1186/s12862-021-01938-y (PMC8591844; doi:10.1186/s12862-021-01938-y)
Supplement: Supplementary file 1 — Additional file 1: Table S1. Test statistics from olfactometer experiments with strains of Lariophagus distinguendus from bird nests and carrion tested on the odour of bird nests or carrion. The odour field contained samples of bird nests or carrion. Strain abbreviations with “N” refer to strains collected in bird nests, strains with “A” were collected next to carrions. Different letters within the same row indicate significant differences in allocation time between fields (p < 0.005). Single comparisons were made using Tukey-test based on linear mixed models or generalized mixed models with field as factor and observation as random factor (Table S2, separate file). Lines with significant differences between odour field and control field 2 are shaded. Table S2. Test statistics from olfactometer experiments with strains of Nasonia vitripennis from bird nests and carrion tested on the odour of bird nests or carrion. Strain abbreviations with “N” refer to strains collected in bird nests, strains with “A” were collected next to carrions. Table S3. Distribution of individuals between main and alternative microhabitat in the different subpopulations identified by microsatellites for k = 3 and k = 5 subpopulations. The main habitats are bird nests for subpopulation 1 and carrion for subpopulations 2–5. Table S4. Mean number (± S.D.) of F1-female offspring from couples consisting of females and males from different strains. Data from intra-strain couples are shaded. [file 12862_2021_1938_MOESM1_ESM.docx]

**Table S1:** Test statistics from olfactometer experiments with strains of Lariophagus distinguendus from bird nests and carrion tested on the odour of bird nests or carrion. The odour field contained samples of bird nests or carrion. Strain abbreviations with “N” refer to strains collected in bird nests, strains with “A” were collected next to carrions. Different letters within the same row indicate significant differences in allocation time between fields (p < 0.005). Single comparisons were made using Tukey-test based on linear mixed models or generalized mixed models with field as factor and observation as random factor (table S2, separate file). Lines with significant differences between odour field and control field 2 are shaded.

|  |  | | | Mean allocation time ± standard deviation in fields (s) | | | | | | | | | |
| --- | --- | --- | --- | --- | --- | --- | --- | --- | --- | --- | --- | --- | --- |
| odour | strain | | | odour field | | | control 1 | | | | control 2 | | control 3 |
| nest | N2 | | | 83±39a | | | 55±21b | | | | 47±24b | | 52±23b |
|  | N3 | | | 99±33a | | | 62±23b | | | | 45±25b | | 47±26b |
|  | N5 | | | 110±50a | | | 69±36ab | | | | 39±27c | | 43±21bc |
|  | N6 | | | 96±45a | | | 60±26b | | | | 38±34c | | 53±21bc |
|  | N9 | | | 93±45a | | | 74±43a | | | | 35±24b | | 41±24b |
|  | N11 | | | 95±31a | | | 60±2b9 | | | | 54±23b | | 65±22b |
|  | N12 | | | 81±32a | | | 66±26ab | | | | 59±29b | | 46±20b |
|  | N15 | | | 91±37a | | | 82±21a | | | | 45±25b | | 45±28b |
|  | N17 | | | 98±46a | | | 59±27b | | | | 45±22b | | 53±27b |
| carrion | N2 | | | 111±57a | | | 55±30b | | | | 40±33b | | 71±29b |
|  | N3 | | | 83±48a | | | 70±41a | | | | 49±38a | | 54±47a |
|  | N5 | | | 94±52a | | | 59±37b | | | | 54±39b | | 52±32b |
|  | N6 | | | 75±42a | | | 86±45a | | | | 40±28b | | 44±26b |
|  | N9 | | | 72±41ab | | | 42±32b | | | | 78±46a | | 60±36ab |
|  | N10 | | | 78±37a | | | 69±33ab | | | | 45±26b | | 67±37ab |
|  | N11 | | | 74±39a | | | 60±26a | | | | 52±20a | | 64±26a |
|  | N12 | | | 67±42a | | | 58±40a | | | | 54±44a | | 62±36a |
|  | N14 | | | 63±35a | | | 60±25a | | | | 69±35a | | 69±27a |
|  | N15 | | | 91±66a | | | 81±45ab | | | | 50±34bc | | 42±33c |
|  | N17 | | | 85±61a | | | 97±57a | | | | 53±41ab | | 32±37b |
| nest | | A1 | | | 67±33a | | | 66±33a | | 61±28a | | | 62±27a |
|  | | A4 | | | 81±36a | | | 70±28a | | 56±28a | | | 56±39a |
|  | | A5 | | | 72±25ab | | | 90±34b | | 45±19c | | | 56±26bc |
|  | | A7 | | | 73±30a | | | 67±22a | | 70±23a | | | 45±22b |
|  | | A8 | | | 76±23a | | | 77±26a | | 65±26ab | | | 50±21b |
|  | | A10 | | | 105±52a | | | 77±47a | | 44±25b | | | 41±25b |
|  | | A14 | | | 75±31a | | | 92±37a | | 43±24b | | | 48±24b |
|  | | A15 | | | 58±28a | | | 75±46a | | 53±24a | | | 55±31a |
|  | | A16 | | | 76±37a | | | 78±31a | | 67±28a | | | 59±24a |
|  | | A19 | | | 54±29a | | | 83±38b | | 62±32ab | | | 53±34a |
| carrion | | | A1 | | | 166±59a | | | 47±30b | | | 17±19c | 26±28c |
|  | | | A4 | | | 170±62a | | | 36±31b | | | 19±26b | 34±28b |
|  | | | A5 | | | 142±86a | | | 50±57b | | | 20±25b | 40±42b |
|  | | | A7 | | | 167±64a | | | 53±49b | | | 17±15c | 25±30bc |
|  | | | A8 | | | 126±48a | | | 53±37b | | | 36±30b | 43±28b |
|  | | | A10 | | | 152±61a | | | 51±35b | | | 17±18c | 51±43b |
|  | | | A14 | | | 171±47a | | | 55±31b | | | 18±20c | 26±21c |
|  | | | A15 | | | 161±45a | | | 52±26b | | | 15±17c | 32±30bc |
|  | | | A16 | | | 124±67a | | | 65±39b | | | 25±30c | 43±32bc |
|  | | | A19 | | | 141±71a | | | 41±36b | | | 19±18b | 37±28b |

**Table S2:** Test statistics from olfactometer experiments with strains of *Nasonia vitripennis* from bird nests and carrion tested on the odour of bird nests or carrion. Strain abbreviations with “N” refer to strains collected in bird nests, strains with “A” were collected next to carrions.

**N2 on nest odour** (Linear mixed model, observation as random factor, n=20)

|  | Estimate | Std. Error | t-value | Pr(>\|z\|) |  |
| --- | --- | --- | --- | --- | --- |
| (Intercept) | 82.810 | 6.185 | 13.388 | <2e-16 | *** |
| fieldfield2 | -27.529 | 8.748 | -3.147 | 0002355 | ** |
| fieldfield3 | -35.908 | 8.748 | -4.105 | 0.000101 | *** |
| fieldfield4 | -30.960 | 8.748 | -3.539 | 0.000688 | *** |

**N3 on nest odour** (Linear mixed model, observation as random factor, n=20)

|  | Estimate | Std. Error | t-value | Pr(>\|z\|) |  |
| --- | --- | --- | --- | --- | --- |
| (Intercept) | 98.534 | 6.074 | 16.222 | <2e-16 | *** |
| fieldfield2 | -36.722 | 8.590 | -4.275 | 5.49e-05 | *** |
| fieldfield3 | -53.870 | 8.590 | -6.271 | 2.00e-08 | *** |
| fieldfield4 | -51.062 | 8.590 | -5.944 | 7.89e-08 | *** |

**N5 on nest odour** (Generalized linear mixed model, observation as random factor (negativ binomial), n=20)

|  | Estimate | Std. Error | z-value | Pr(>\|z\|) |  |
| --- | --- | --- | --- | --- | --- |
| (Intercept) | 4.6969 | 0.1401 | 33.535 | <2e-16 | *** |
| fieldfield2 | -0.4610 | 0.1987 | -2.319 | 0.0204 | * |
| fieldfield3 | -1.0252 | 0.2001 | -5.123 | 3.01e-07 | *** |
| fieldfield4 | -0.9364 | 0.1999 | -4.685 | 2.80e-06 | *** |

**N6 on nest odour** (Linear mixed model, observation as random factor (sqrt-transformed), n=20)

|  | Estimate | Std. Error | t-value | Pr(>\|z\|) |  |
| --- | --- | --- | --- | --- | --- |
| (Intercept) | 9.4882 | 0.4938 | 19.213 | <2e-16 | *** |
| fieldfield2 | -1.8842 | 0.6984 | -2.698 | 0.008592 | ** |
| fieldfield3 | -3.9345 | 0.6984 | -5.634 | 2.85e-07 | *** |
| fieldfield4 | -2.4080 | 0.6984 | -3.448 | 0.000924 | *** |

**N7 on nest odour** (Linear mixed model, observation as random factor (sqrt-transformed), n=20)

|  | Estimate | Std. Error | t-value | Pr(>\|z\|) |  |
| --- | --- | --- | --- | --- | --- |
| (Intercept) | 9.3640 | 0.4383 | 21.363 | <2e-16 | *** |
| fieldfield2 | -0.9640 | 0.6199 | -1.555 | 0.124 |  |
| fieldfield3 | -3.0259 | 0.6199 | -4.881 | 5.68e-06 | *** |
| fieldfield4 | -3.1603 | 0.6199 | -5.098 | 2.44e-06 | *** |

**N8 on nest odour** (Linear mixed model, observation as random factor as random factor , n=20)

|  | Estimate | Std. Error | t-value | Pr(>\|z\|) |  |
| --- | --- | --- | --- | --- | --- |
| (Intercept) | 87.576 | 6.206 | 14.111 | <2e-16 | *** |
| fieldfield2 | -13.146 | 8.777 | -1.498 | 0.138 |  |
| fieldfield3 | -45.841 | 8.777 | -5.223 | 1.49e-06 | *** |
| fieldfield4 | -39.310 | 8.777 | -4.479 | 2.60e-05 | *** |

**N9 on nest odour** (Linear mixed model, observation as random factor as random factor , n=20)

|  | Estimate | Std. Error | t-value | Pr(>\|z\|) |  |
| --- | --- | --- | --- | --- | --- |
| (Intercept) | 93.508 | 7.913 | 11.816 | <2e-16 | *** |
| fieldfield2 | -19.232 | 11.191 | -1.719 | 0.0898 | . |
| fieldfield3 | -58.517 | 11.191 | -5.229 | 1.46e-06 | *** |
| fieldfield4 | -52.514 | 11.191 | -4.692 | 1.17e-05 | *** |

**N11 on nest odour** (Linear mixed model, observation as random factor, n=20)

|  | Estimate | Std. Error | t-value | Pr(>\|z\|) |  |
| --- | --- | --- | --- | --- | --- |
| (Intercept) | 94.754 | 5.928 | 15.984 | <2e-16 | *** |
| fieldfield2 | .34.580 | 8.383 | -4.125 | 9.39e-05 | *** |
| fieldfield3 | -40.389 | 8.383 | -4.818 | 7.26e-06 | *** |
| fieldfield4 | -29.540 | 8.383 | -3.524 | 0.000724 | *** |

**N12 on nest odour** (Linear mixed model, observation as random factor, n=20)

|  | Estimate | Std. Error | t-value | Pr(>\|z\|) |  |
| --- | --- | --- | --- | --- | --- |
| (Intercept) | 81.078 | 6.018 | 13.472 | <2e-16 | *** |
| fieldfield2 | -15.203 | 8.511 | -1.786 | 0.078062 | . |
| fieldfield3 | -22.407 | 8.511 | -2.633 | 0.010260 | * |
| fieldfield4 | -34.694 | 8.511 | -4.076 | 0.000112 | *** |

**N13 on nest odour** (Linear mixed model, observation as random factor, n=20)

|  | Estimate | Std. Error | t-value | Pr(>\|z\|) |  |
| --- | --- | --- | --- | --- | --- |
| (Intercept) | 101.991 | 5.973 | 17.076 | <2e-16 | *** |
| fieldfield2 | -42.407 | 8.447 | -5.020 | 3.31e-06 | *** |
| fieldfield3 | -69.950 | 8.447 | -8.281 | 3.19e-12 | *** |
| fieldfield4 | -41.675 | 8.447 | -4.934 | 4.64-06 | *** |

**N15 on nest odour** (Linear mixed model, observation as random factor (sqrt-transformed), n=20)

|  | Estimate | Std. Error | t-value | Pr(>\|z\|) |  |
| --- | --- | --- | --- | --- | --- |
| (Intercept) | 9.3739 | 0.4405 | 21.282 | <2e-16 | *** |
| fieldfield2 | -0.3813 | 0.6229 | -0.612 | 0.542 |  |
| fieldfield3 | -3.0758 | 0.6229 | -4.938 | 4.56e-06 | *** |
| fieldfield4 | -3.0398 | 0.6229 | -4.880 | 5.71e-06 | *** |

**N17 on nest odour** (Linear mixed model, observation as random factor, n=20)

|  | Estimate | Std. Error | t-value | Pr(>\|z\|) |  |
| --- | --- | --- | --- | --- | --- |
| (Intercept) | 97.566 | 7.107 | 13.728 | <2e-16 | *** |
| fieldfield2 | -38.834 | 10.051 | -3.864 | 0.000233 | *** |
| fieldfield3 | -52.652 | 10.051 | -5.238 | 1.40e-06 | *** |
| fieldfield4 | -44.583 | 10.051 | -4.436 | 3.05e-05 | *** |

**Strain N2 on carrion odour** (Linear mixed model, observation as random factor, n=20)

|  | Estimate | Std. Error | t-value | Pr(>\|z\|) |  |
| --- | --- | --- | --- | --- | --- |
| (Intercept) | 111.030 | 8.812 | 12.600 | <2e-16 | *** |
| fieldfield2 | -56.018 | 12.462 | -4.495 | 2.45e-05 | *** |
| fieldfield3 | -70.765 | 12.462 | -5.678 | 2.37e-07 | *** |
| fieldfield4 | -40.229 | 12.462 | -3.228 | 0.00184 | ** |

**Strain N3 on carrion odour** (Linear mixed model, observation as random factor (sqrt-transformed), n=20)

|  | Estimate | Std. Error | t-value | Pr(>\|z\|) |  |
| --- | --- | --- | --- | --- | --- |
| (Intercept) | 8.5577 | 0.7181 | 11.917 | <2e-16 | *** |
| fieldfield2 | -0.6434 | 1.0156 | -0.634 | 0.5283 |  |
| fieldfield3 | -2.3085 | 1.0156 | -2.273 | 0.0258 | * |
| fieldfield4 | -2.0619 | 1.0156 | -2.030 | 0.0458 | * |

**Strain N5 on carrion odour** (Linear mixed model, observation as random factor, n=20)

|  | Estimate | Std. Error | t-value | Pr(>\|z\|) |  |
| --- | --- | --- | --- | --- | --- |
| (Intercept) | 93.589 | 9.172 | 10.204 | 6.84e-16 | *** |
| fieldfield2 | -34.999 | 12.971 | -2.698 | 0.00858 | ** |
| fieldfield3 | -39.291 | 12.971 | -3.029 | 0.00335 | ** |
| fieldfield4 | -41.203 | 12.971 | -3.176 | 0.00215 | ** |

**Strain N6 on carrion odour** (Linear mixed model, observation as random factor, n=20)

|  | Estimate | Std. Error | t-value | Pr(>\|z\|) |  |
| --- | --- | --- | --- | --- | --- |
| (Intercept) | 74.774 | 8.041 | 9.299 | 3.58e-14 | *** |
| fieldfield2 | 11.298 | 11.372 | 0.993 | 0.32362 |  |
| fieldfield3 | -34.341 | 11.372 | -3.020 | 0.00344 | ** |
| fieldfield4 | -31.047 | 11.372 | -2.730 | 0.00786 | ** |

**Strain N7 on carrion odour** (Linear mixed model, observation as random factor (sqrt-transformed), n=20)

|  | Estimate | Std. Error | t-value | Pr(>\|z\|) |  |
| --- | --- | --- | --- | --- | --- |
| (Intercept) | 9.9587 | 0.5819 | 17.115 | <2e-16 | *** |
| fieldfield2 | -1.8931 | 0.8229 | -2.301 | 0.0242 | * |
| fieldfield3 | -4.6391 | 0.8229 | -5.638 | 2.80e-07 | *** |
| fieldfield4 | -3.6447 | 0.8229 | -4.429 | 3.13e-05 | *** |

**Strain N8 on carrion odour** (Linear mixed model, observation as random factor (sqrt-transformed), n=20)

|  | Estimate | Std. Error | t-value | Pr(>\|z\|) |  |
| --- | --- | --- | --- | --- | --- |
| (Intercept) | 8.8889 | 0.6353 | 13.992 | <2e-16 | *** |
| fieldfield2 | -0.7813 | 0.8984 | -0.870 | 0.3870 |  |
| fieldfield3 | -2.4804 | 0.8984 | -2.761 | 0.00722 | ** |
| fieldfield4 | -2.3241 | 0.8984 | -2.587 | 0.01160 | * |

**Strain N9 on carrion odour** (Linear mixed model, observation as random factor, n=20)

|  | Estimate | Std. Error | t-value | Pr(>\|z\|) |  |
| --- | --- | --- | --- | --- | --- |
| (Intercept) | 72.069 | 8.811 | 8.179 | 5e-12 | *** |
| fieldfield2 | -30.027 | 12.461 | -2.410 | 0.0184 | * |
| fieldfield3 | 6.026 | 12.461 | 0.484 | 0.6301 |  |
| fieldfield4 | -12.439 | 12.461 | -0.998 | 0.3214 |  |

**Strain N10 on carrion odour** (Linear mixed model, observation as random factor, n=20)

|  | Estimate | Std. Error | t-value | Pr(>\|z\|) |  |
| --- | --- | --- | --- | --- | --- |
| (Intercept) | 77.749 | 7.493 | 10.376 | 3.24e-16 | *** |
| fieldfield2 | -8.681 | 10.597 | -0.819 | 0.41521 |  |
| fieldfield3 | -32.488 | 10.597 | -3.066 | 0.00301 | ** |
| fieldfield4 | -10.244 | 10.597 | -0.967 | 0.33677 |  |

**Strain N11 on carrion odour** (Generalized linear mixed model, observation as random factor (negative binomial), n=20)

|  | Estimate | Std. Error | z-value | Pr(>\|z\|) |  |
| --- | --- | --- | --- | --- | --- |
| (Intercept) | 4.2996 | 0.1098 | 39.152 | <2e-16 | *** |
| fieldfield2 | -0.2039 | 0.1558 | -1.309 | 0.1906 |  |
| fieldfield3 | -0.3435 | 0.1562 | -2.199 | 0.0279 | * |
| fieldfield4 | -0.1328 | 0.1556 | -0.854 | 0.3933 |  |

**Strain N12 on carrion odour** (Linear mixed model, observation as random factor (sqrt-transformed), n=20)

|  | Estimate | Std. Error | t-value | Pr(>\|z\|) |  |
| --- | --- | --- | --- | --- | --- |
| (Intercept) | 7.8976 | 0.6298 | 12.539 | <2e-16 | *** |
| fieldfield2 | -0.9216 | 0.8907 | -1.035 | 0.304 |  |
| fieldfield3 | -1.2875 | 0.8907 | -1.445 | 0.152 |  |
| fieldfield4 | -0.4289 | 0.8907 | -0.481 | 0.632 |  |

**Strain N13 on carrion odour** (Generalized linear mixed model, observation as random factor (negative binomial), n=20)

|  | Estimate | Std. Error | z-value | Pr(>\|z\|) |  |
| --- | --- | --- | --- | --- | --- |
| (Intercept) | 4.02773 | 0.14602 | 27.584 | <2e-16 | *** |
| fieldfield2 | 0.33659 | 0.20588 | 1.635 | 0.102 |  |
| fieldfield3 | 0.04316 | 0.20641 | 0.209 | 0.834 |  |
| fieldfield4 | -0.01645 | 0.20654 | -0.080 | 0.937 |  |

**Strain N14 on carrion odour** (Linear mixed model, observation as random factor, n=20)

|  | Estimate | Std. Error | t-value | Pr(>\|z\|) |  |
| --- | --- | --- | --- | --- | --- |
| (Intercept) | 63.165 | 6.914 | 9.136 | 7.33e-14 | *** |
| fieldfield2 | -3.405 | 9.778 | -0.348 | 0.729 |  |
| fieldfield3 | 5.675 | 9.778 | 0.580 | 0.563 |  |
| fieldfield4 | 6.177 | 9.778 | 0.632 | 0.526 |  |

**Strain N15 on carrion odour** (Linear mixed model, observation as random factor, n=20)

|  | Estimate | Std. Error | t-value | Pr(>\|z\|) |  |
| --- | --- | --- | --- | --- | --- |
| (Intercept) | 90.710 | 10.403 | 8.720 | 4.6e-13 | *** |
| fieldfield2 | -9.857 | 14.712 | -0.670 | 0.50491 |  |
| fieldfield3 | -40.722 | 14.712 | -2.768 | 0.00708 | ** |
| fieldfield4 | -49.019 | 14.712 | -3.332 | 0.00133 | ** |

**Strain N17 on carrion odour** (Linear mixed model, observation as random factor (sqrt-transformed), n=20)

|  | Estimate | Std. Error | t-value | Pr(>\|z\|) |  |
| --- | --- | --- | --- | --- | --- |
| (Intercept) | 8.5566 | 0.7402 | 11.559 | <2e-16 | *** |
| fieldfield2 | 0.6741 | 1.0468 | 0.644 | 0.52157 |  |
| fieldfield3 | -1.7016 | 1.0468 | -1.625 | 0.10820 |  |
| fieldfield4 | -4.0630 | 1.0468 | -3.881 | 0.00022 | *** |

**Strain A1 on nest odour** (Linear mixed model, observation as random factor, n=20)

|  | Estimate | Std. Error | t-value | Pr(>\|z\|) |  |
| --- | --- | --- | --- | --- | --- |
| (Intercept) | 67.211 | 6.787 | 9.903 | 2.54e-15 | *** |
| fieldfield2 | -1.491 | 9.599 | -0.155 | 0.877 |  |
| fieldfield3 | -6.037 | 9.599 | -0.629 | 0.531 |  |
| fieldfield4 | -5.048 | 9.599 | -0.526 | 0.600 |  |

**Strain A4 on nest odour** (Generalized linear mixed model, observation as random factor (negative binomial), n=20)

|  | Estimate | Std. Error | z-value | Pr(>\|z\|) |  |
| --- | --- | --- | --- | --- | --- |
| (Intercept) | 4.3957 | 0.1182 | 37.184 | <2e-16 | *** |
| fieldfield2 | -0.1441 | 0.1675 | -0.860 | 0.3897 |  |
| fieldfield3 | -0.3759 | 0.1680 | -2.237 | 0.0253 | * |
| fieldfield4 | -0.3621 | 0.1680 | -2.156 | 0.0311 | * |

**Strain A5 on nest odour** (Linear mixed model, observation as random factor, n=20)

|  | Estimate | Std. Error | t-value | Pr(>\|z\|) |  |
| --- | --- | --- | --- | --- | --- |
| (Intercept) | 72.228 | 5.940 | 12.159 | <2e-16 | *** |
| fieldfield2 | 17.455 | 8.401 | 2.078 | 0.04111 | * |
| fieldfield3 | -27.298 | 8.401 | -3.249 | 0.00172 | ** |
| fieldfield4 | -16.227 | 8.401 | -1.932 | 0.05714 | . |

**Strain A7 on nest odour** (Linear mixed model, observation as random factor, n=20)

|  | Estimate | Std. Error | t-value | Pr(>\|z\|) |  |
| --- | --- | --- | --- | --- | --- |
| (Intercept) | 73.106 | 5.449 | 13.417 | <2e-16 | *** |
| fieldfield2 | -6.413 | 7.705 | -0.832 | 0.407901 |  |
| fieldfield3 | -2.872 | 7.705 | -0.832 | 0.710392 |  |
| fieldfield4 | -27.583 | 7.705 | -3.580 | 0.000604 | *** |

**Strain A8 on nest odour** (Linear mixed model, observation as random factor, n=20)

|  | Estimate | Std. Error | t-value | Pr(>\|z\|) |  |
| --- | --- | --- | --- | --- | --- |
| (Intercept) | 75.651 | 5.369 | 14.089 | <2e-16 | *** |
| fieldfield2 | 1.551 | 7.593 | 0.204 | 0.83875 |  |
| fieldfield3 | -10.167 | 7.593 | -1.339 | 0.18457 |  |
| fieldfield4 | -25.209 | 7.593 | -3.320 | 0.00139 | ** |

**Strain A10 on nest odour** (Linear mixed model, observation as random factor (sqrt-transformed), n=20)

|  | Estimate | Std. Error | t-value | Pr(>\|z\|) |  |
| --- | --- | --- | --- | --- | --- |
| (Intercept) | 9.9785 | 0.5259 | 18.974 | <2e-16 | *** |
| fieldfield2 | -1.5745 | 0.7437 | -2.117 | 0.0375 | * |
| fieldfield3 | -3.7465 | 0.7437 | -5.037 | 3.10e-06 | *** |
| fieldfield4 | -3.9015 | 0.7437 | -5.246 | 1.36e-06 | *** |

**Strain A14 on nest odour** (Linear mixed model, observation as random factor (sqrt-transformed), n=20)

|  | Estimate | Std. Error | t-value | Pr(>\|z\|) |  |
| --- | --- | --- | --- | --- | --- |
| (Intercept) | 8.4854 | 0.3997 | 21.230 | <2e-16 | *** |
| fieldfield2 | 0.9502 | 0.5652 | 1.681 | 0.096866 | . |
| fieldfield3 | -2.1513 | 0.5652 | -3.806 | 0.000284 | *** |
| fieldfield4 | -1.7502 | 0.5652 | -3.096 | 0.002743 | ** |

**Strain A15 on nest odour** (Linear mixed model, observation as random factor (sqrt-transformed), n=20)

|  | Estimate | Std. Error | t-value | Pr(>\|z\|) |  |
| --- | --- | --- | --- | --- | --- |
| (Intercept) | 7.3905 | 0.4795 | 15.412 | <2e-16 | *** |
| fieldfield2 | 0.8513 | 0.6782 | 1.255 | 0.213 |  |
| fieldfield3 | -0.3528 | 0.6782 | -0.520 | 0.605 |  |
| fieldfield4 | -0.2810 | 0.6782 | -0.414 | 0.680 |  |

**Strain A16 on nest odour** (Linear mixed model, observation as random factor, n=20)

|  | Estimate | Std. Error | t-value | Pr(>\|z\|) |  |
| --- | --- | --- | --- | --- | --- |
| (Intercept) | 76.374 | 6.829 | 11.184 | <2e-16 | *** |
| fieldfield2 | 1.553 | 9.658 | 0.161 | 0.8727 |  |
| fieldfield3 | -8.868 | 9.658 | -0.918 | 0.3614 |  |
| fieldfield4 | -17.229 | 9.658 | -1.784 | 0.0784 | . |

**Strain A19 on nest odour** (Linear mixed model, observation as random factor, n=20)

|  | Estimate | Std. Error | t-value | Pr(>\|z\|) |  |
| --- | --- | --- | --- | --- | --- |
| (Intercept) | 54.320 | 7.476 | 7.266 | 2.77e-10 | *** |
| fieldfield2 | 28.959 | 10.573 | 2.739 | 0.00768 | ** |
| fieldfield3 | 7.399 | 10.573 | 0.700 | 0.48620 |  |
| fieldfield4 | -1.687 | 10.573 | -0.160 | 0.87362 |  |

**Strain A1 on carrion odour** (Linear mixed model, observation as random factor (sqrt-transformed), n=20)

|  | Estimate | Std. Error | t-value | Pr(>\|z\|) |  |
| --- | --- | --- | --- | --- | --- |
| (Intercept) | 12.6951 | 0.5996 | 21.279 | <2e-16 | *** |
| fieldfield2 | -6.2073 | 0.8437 | -7.357 | 1.85e-10 | *** |
| fieldfield3 | -9.5718 | 0.8437 | -11.345 | <2e-16 | *** |
| fieldfield4 | -8.4418 | 0.8437 | -10.005 | 1.62e-15 | *** |

**Strain A4 on carrion odour** (Linear mixed model, observation as random factor, n=20)

|  | Estimate | Std. Error | t-value | Pr(>\|z\|) |  |
| --- | --- | --- | --- | --- | --- |
| (Intercept) | 170.461 | 8.909 | 19.13 | <2e-16 | *** |
| fieldfield2 | -134.019 | 12.599 | -10.64 | <2e-16 | *** |
| fieldfield3 | -151.328 | 12.599 | -12.01 | <2e-16 | *** |
| fieldfield4 | -136.847 | 12.599 | -10.86 | <2e-16 | *** |

**Strain A5 on carrion odour** (Generalized linear mixed model, observation as random factor (negative binomial), n=20)

|  | Estimate | Std. Error | t-value | Pr(>\|z\|) |  |
| --- | --- | --- | --- | --- | --- |
| (Intercept) | 4.9550 | 0.2685 | 18.453 | <2e-16 | *** |
| fieldfield2 | -1.0456 | 0.3806 | -2.747 | 0.006007 | ** |
| fieldfield3 | -1.9357 | 0.3825 | -5.061 | 4.17e-07 | *** |
| fieldfield4 | -1.2656 | 0.3809 | -3.322 | 0.000892 | *** |

**Strain A7 on carrion odour** (Linear mixed model, observation as random factor (sqrt-transformed), n=20)

|  | Estimate | Std. Error | t-value | Pr(>\|z\|) |  |
| --- | --- | --- | --- | --- | --- |
| (Intercept) | 12.6909 | 0.6606 | 19.212 | <2e-16 | *** |
| fieldfield2 | -6.4819 | 0.9342 | -6.938 | 1.15e-09 | *** |
| fieldfield3 | -9.2233 | 0.9342 | -9.873 | 2.89e-15 | *** |
| fieldfield4 | -8.5217 | 0.9342 | -9.122 | 7.80e-14 | *** |

**Strain A8 on carrion odour** (Linear mixed model, observation as random factor, n=20)

|  | Estimate | Std. Error | t-value | Pr(>\|z\|) |  |
| --- | --- | --- | --- | --- | --- |
| (Intercept) | 126.506 | 8.191 | 15.444 | <2e-16 | *** |
| fieldfield2 | -73.913 | 11.584 | -6.381 | 1.26e-0.8 | *** |
| fieldfield3 | -90.327 | 11.584 | -7.797 | 2.69e-11 | *** |
| fieldfield4 | -83.939 | 11.584 | -7.246 | 3.01e-10 | *** |

**Strain A10 on carrion odour** (Linear mixed model, observation as random factor (sqrt-transformed), n=20)

|  | Estimate | Std. Error | t-value | Pr(>\|z\|) |  |
| --- | --- | --- | --- | --- | --- |
| (Intercept) | 12.0787 | 0.6138 | 19.679 | <2e-16 | *** |
| fieldfield2 | -5.3983 | 0.8680 | -6.219 | 2.49e-0.8 | *** |
| fieldfield3 | -8.9794 | 0.8680 | -10.345 | 3.71e-16 | *** |
| fieldfield4 | -5.6538 | 0.8680 | -6.513 | 7.14e-09 | *** |

**Strain A14 on carrion odour** (Linear mixed model, observation as random factor, n=20)

|  | Estimate | Std. Error | t-value | Pr(>\|z\|) |  |
| --- | --- | --- | --- | --- | --- |
| (Intercept) | 170.775 | 7.078 | 24.13 | <2e-16 | *** |
| fieldfield2 | -115.930 | 10.010 | -11.58 | <2e-16 | *** |
| fieldfield3 | -153.045 | 10.010 | -15.29 | <2e-16 | *** |
| fieldfield4 | -144.450 | 10.010 | -14.43 | <2e-16 | *** |

**Strain A15 on carrion odour** (Linear mixed model, observation as random factor, n=20)

|  | Estimate | Std. Error | t-value | Pr(>\|z\|) |  |
| --- | --- | --- | --- | --- | --- |
| (Intercept) | 160.809 | 6.966 | 23.09 | <2e-16 | *** |
| fieldfield2 | -109.199 | 9.851 | -11.09 | <2e-16 | *** |
| fieldfield3 | -145.445 | 9.851 | -14.77 | <2e-16 | *** |
| fieldfield4 | -128.755 | 9.851 | -13.07 | <2e-16 | *** |

**Strain A16 on carrion odour** (Linear mixed model, observation as random factor (sqrt-transformed), n=20)

|  | Estimate | Std. Error | t-value | Pr(>\|z\|) |  |
| --- | --- | --- | --- | --- | --- |
| (Intercept) | 10.7687 | 0.6655 | 16.181 | <2e-16 | *** |
| fieldfield2 | -3.2262 | 0.9412 | -3.428 | 0.000985 | *** |
| fieldfield3 | -6.7318 | 0.9412 | -7.153 | 4.52e-10 | *** |
| fieldfield4 | -4.8369 | 0.9412 | -5.139 | 2.08e-06 | *** |

**Strain A19 on carrion odour** (Linear mixed model, observation as random factor (sqrt-transformed), n=20)

|  | Estimate | Std. Error | t-value | Pr(>\|z\|) |  |
| --- | --- | --- | --- | --- | --- |
| (Intercept) | 11.4588 | 0.6525 | 17.526 | <2e-16 | *** |
| fieldfield2 | -5.9194 | 0.9227 | -6.415 | 1.09e-08 | *** |
| fieldfield3 | -7.8944 | 0.9227 | -8.556 | 9.49e-13 | *** |
| fieldfield4 | -5.9726 | 0.9227 | -6.473 | 8.49e-09 | *** |

**Table S3:** Distribution of individuals between main and alternative microhabitat in the different subpopulations identified by microsatellites for k=3 and k=5 subpopulations. The main habitats are bird nests for subpopulation 1 and carrion for subpopulations 2-5.

|  |  | Subpop.  1 | Subpop.  2 | Subpop.  3 | Subpop.  4 | Subpop.  5 | *p^1^* |
| --- | --- | --- | --- | --- | --- | --- | --- |
| k=3 | Main habitat | 70 | 10 | 6 | - | - | 0.3298 |
|  | Alternative habitat | 13 | 1 | 1 | - | - |  |
| k=5 | Main habitat | 70 | 4 | 6 | 4 | 4 | 1 |
|  | Alternative habitat | 10 | 2 | 2 | 0 | 0 |  |

^1^Fisher Exact test.

**Table S4:** Mean number (± S.D.) of F1-female offspring from couples consisting of females and males from different strains. Data from intra-strain couples are shaded.

| female strain | male strain | | | | | |
| --- | --- | --- | --- | --- | --- | --- |
|  | A1 | A7 | A19 | N2 | N3 | N9 |
| A1 | 68.1 ± 25.8 | 30.1±21.4 | 91.1±34.9 | 53.4±37.9 | 35.3±16.1 | 47.5 ±28.2 |
| A7 | 36.5 ± 22.9 | 83.9±28.8 | 91.4±31.9 | 41.5±19.5 | 26.7±18.3 | 22.7 ±10.4 |
| A19 | 79.3 ± 32.3 | 62.2 ±25.7 | 88.4 ±22.0 | 26.9 ±9.9 | 22.6 ±9.6 | 66.3 ±38.0 |
| N2 | 48.8 ± 15.5 | 76.5±19.1 | 49.7 ±19.4 | 71.4 ±28.3 | 35.7 ±16.0 | 77.0 ±23.3 |
| N3 | 19.5 ±14.6 | 53.7 ±34.7 | 32.1 ±13.7 | 29.2 ±10.8 | 41.9 ±21.4 | 28.0 ±10.0 |
| N9 | 42.5 ± 17.5 | 57.2 ±24.0 | 63.2 ±29.3 | 53.9 ±37-6 | 28.1 ±17.3 | 92.5 ±14.1 |
