# Supplementary material for: The emergence of ecotypes in a parasitoid wasp: a case of incipient sympatric speciation in Hymenoptera?
Source: BMC Ecol Evol. 2021 Nov 15;21:204. doi: 10.1186/s12862-021-01938-y (PMC8591844; doi:10.1186/s12862-021-01938-y)
Supplement: Supplementary file 2 — Additional file 2: Fig. S1. Box and whisker plot of pupae of Lucilia sericata carrion flies parasitized by Nasonia vitripennis wasps. N2, N3, N9: Wasp strains that were collected in bird nests (yellow, n = 30 per strain). A1, A7, A19 Wasp strains that were collected in next to carrion (blue, n = 30 per strain). The plots show minimum, maximum, 1st and 3rd quartile, median, outliers as circles and mean as asterisk. Fig. S2. Box and whisker plot of offspring emerging from pupae of Lucilia sericata carrion flies parasitized by Nasonia vitripennis wasps. N2, N3, N9: Wasp strains that were collected in bird nests (yellow, n = 30 per strain). A1, A7, A19 Wasp strains that were collected in next to carrion (blue, n = 30 per strain). The plots show minimum, maximum, 1st and 3rd quartile, median, outliers as circles and mean as asterisk. Fig. S3. Putative population structure of the parasitoid N. vitripennis in the Hohenheim Park (Germany) based on Delta K. Results from three microsatellite analyses (Run 1-Run 3) using STRUCTURE v2.3.4 [159], and the online tool CLUMPAK. [file 12862_2021_1938_MOESM2_ESM.docx]

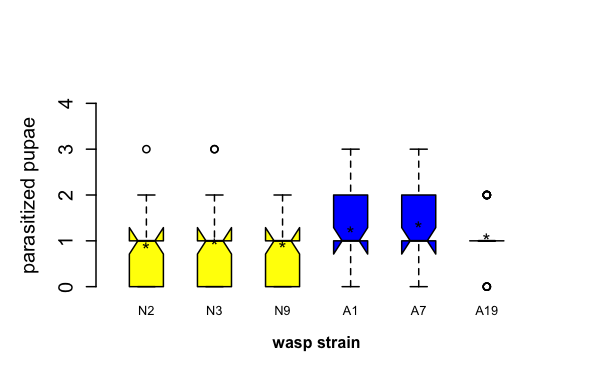


**Fig. S1:** Box and whisker plot of pupae of Lucilia sericata carrion flies parasitized by Nasonia vitripennis wasps. N2, N3, N9: Wasp strains that were collected in bird nests (yellow, n=30 per strain). A1, A7, A19 Wasp strains that were collected in next to carrion (blue, n=30 per strain). The plots show minimum, maximum, 1^st^ and 3^rd^ quartile, median, outliers as circles and mean as asterisk.

**
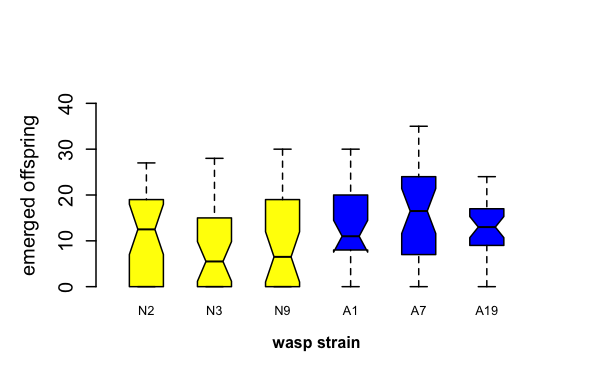
**

**Fig. S2:** Box and whisker plot of offspring emerging from pupae of Lucilia sericata carrion flies parasitized by Nasonia vitripennis wasps. N2, N3, N9: Wasp strains that were collected in bird nests (yellow, n=30 per strain). A1, A7, A19 Wasp strains that were collected in next to carrion (blue, n=30 per strain). The plots show minimum, maximum, 1^st^ and 3^rd^ quartile, median, outliers as circles and mean as asterisk.

**Figure S3:** Putative population structure of the parasitoid *N. vitripennis* in the Hohenheim Park (Germany) based on Delta K. Results from three microsatellite analyses (Run 1-Run 3) using STRUCTURE v2.3.4 [133], and the online tool CLUMPAK (Van Oosterhout *et al.*, 2004).
